# A pseudoenzyme enables indole biosynthesis in eudicot plants

**DOI:** 10.1038/s41589-025-01943-y

**Published:** 2025-06-25

**Authors:** Matilde Florean, Hedwig Schultz, Veit Grabe, Katrin Luck, Grit Kunert, Sarah E. O’Connor, Tobias G. Köllner

**Affiliations:** 1 Department of Natural Product Biosynthesis, Max Planck Institute for Chemical Ecology, Jena, Germany; 2 Microscopic Imaging Service Group, Max Planck Institute for Chemical Ecology, Jena, Germany; 3 Department of Biochemistry, Max Planck Institute for Chemical Ecology, Jena, Germany

**Keywords:** Enzymes, Plant sciences, Biosynthesis, Natural products

## Abstract

Indole is an important biomolecule in plants, essential for amino acid biosynthesis, defense, pollinator attraction and plant–plant communication. Its biosynthesis is reported to be catalyzed by standalone indole-3-glycerol phosphate lyases, which are, however, absent in core eudicots. Here we show that, in core eudicots, indole production for defense and signaling occurs through an alternative pathway. The tryptophan synthase α subunit (TSA), which is typically complexed with the β subunit (TSB) to synthesize tryptophan through indole as an intermediate, can be hijacked by a noncatalytic paralog of TSB (TSB-like) to produce free indole. TSB-like is a pseudoenzyme that evolved from TSB by mutagenesis of two key essential residues, retaining the ability to allosterically activate TSA to allow formation and release of indole. The widespread occurrence and expression pattern of TSB-like genes in plants suggest that this is a general mechanism for the formation of free indole in plant defense and communication.

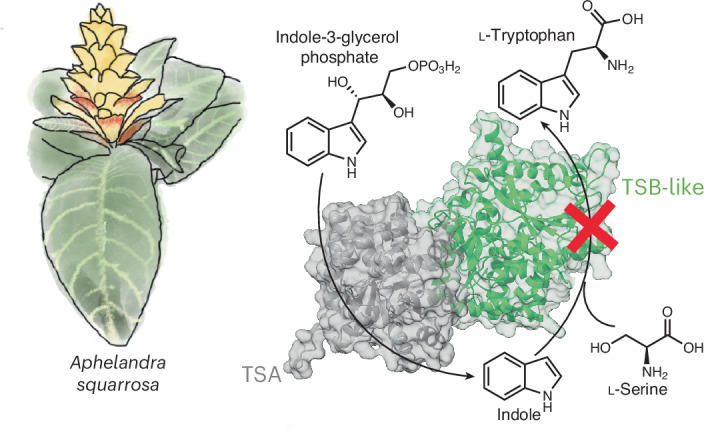

## Abstract (web summary)

Despite the importance of indole in plant defense and communication, its biosynthesis in core eudicots remains elusive. Here the authors report the identification of TSB-like, a pseudoenzyme that hijacks an enzyme of tryptophan biosynthesis to produce indole.

## Main

**Fig. 1:**
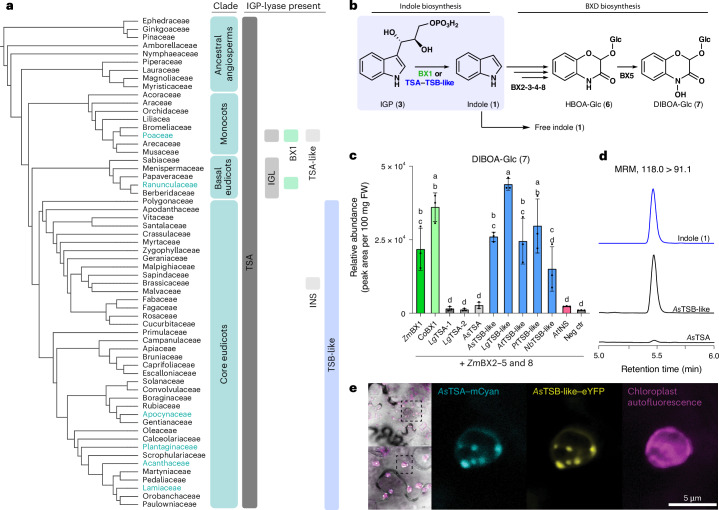
Plants have several ways to produce indole. **a**, Occurrence of BXD-producing species and indole biosynthetic enzymes among plant families. Phylogenetic tree of plant families, based on the Kew Gardens tree of life dataset^54^. Plant families comprising BXD-producing species are colored in green. Types of IGP lyase enzymes present are indicated on the side. **b**, Indole as a precursor for BXDs is produced by BX1 in monocots and basal eudicots or, as reported here, by TSA–TSB-like complexes in core eudicots. BX2, BX3, BX4, BX5 and BX8 enzymes convert indole into the BXD HBOA-Glc and DIBOA-Glc. **c**, BX1 and TSB-like from different species provide indole as a precursor for BXDs. *Bx1* genes from *Z.* *mays* and *C.* *orientalis*, TSA genes from *L.* *galeobdolon* and *A.* *squarrosa*, *TSB-like* genes from *A.* *squarrosa*, *L.* *galeobdolon*, *P.* *trichocarpa*, *A.* *thaliana* and *N.* *benthamiana* and the *INS* gene from *A.* *thaliana* were transiently expressed in *N.* *benthamiana* along with the BXD biosynthetic genes *Bx2*, *Bx3*, *Bx4*, *Bx5* and *Bx8* from *Z.* *mays*. The negative control (neg ctr) consisted of lines expressing only *Bx2*, *Bx3*, *Bx4*, *Bx5* and *Bx8*. Bar graphs represent the mean ± s.d. for three independent biological replicates (*n* = 3 plants). Columns labeled with different letters represent statistically significant differences (*P* < 0.05, one-way analysis of variance (ANOVA) with Tukey’s correction for multiple comparisons). FW, fresh weight. **d**, *As*TSB-like promotes the formation of indole. MRM showing indole accumulation upon transient expression of *AsTSB-like* or *AsTSA* in *N.* *benthamiana*. The chromatogram scale (*y* axis, peak intensity) is equalized across all chromatograms. **e**, *As*TSA and *As*TSB-like transiently expressed in *N.* *benthamiana* colocalize in the chloroplast. *As*TSA–mCyan, *As*TSB-like–eYFP and chloroplast autofluorescence are displayed. Left: larger sections of bright field images with superimposed fluorescence channels. Dashed box (top) indicates dimensions of bottom image. Dashed box (bottom) indicates dimensions of fluorescence images. Source data

Indole (**1**) is a nitrogen-containing aromatic compound that functions as a central intermediate in the biosynthesis of the amino acid tryptophan (**2**) in all forms of life. In several plants, indole also serves as a precursor for the biosynthesis of specialized defense metabolites, including benzoxazinoids (BXDs)^1^ (Fig. 1a,b), nudicaulins^2^ and indigoids^3^. Moreover, many plants release volatile indole upon herbivory to either deter the herbivore or warn neighboring plants of impending attack, thereby priming plant resistance^4–12^. Indole is also released by several plant species as a flower volatile that is involved in attracting pollinators^13–15^.

The formation of indole for various biological processes in plants is carried out by different types of indole-3-glycerol phosphate (IGP) lyases, all of which catalyze the retro-aldol cleavage of IGP (**3**) to indole and glyceraldehyde-3-phosphate but differ in their allosteric activation requirements and heteromeric state^16,17^. The tryptophan synthase α subunit (TSA) is a ubiquitous IGP lyase that produces indole as an intermediate of tryptophan biosynthesis in all kingdoms of life, including plants (Fig.1a)^16–19^. Indole produced by TSA is channeled into the active site of the tryptophan synthase β-subunit (TSB), where it is condensed with l-serine in a pyridoxal phosphate (PLP)-dependent manner to form l-tryptophan^20–22^. TSA and TSB alone have low catalytic activity; however, the formation of a heterotetrameric αββα complex provides a mutual allosteric activation that is required for both of these enzymes to work efficiently^21–23^. Binding of IGP to TSA triggers allosteric activation of TSB, which in turn promotes IGP cleavage and indole biosynthesis in the TSA subunit. This coordinated activation mechanism of the TSA–TSB complex prevents the release of indole from the complex^24^ and, thus, its emission as a volatile or its conversion into downstream specialized metabolites. To produce indole for volatile emission or as a precursor for the biosynthesis of specialized metabolites, plants have evolved two types of standalone IGP lyases, namely IGL and benzoxazinoneless 1 (BX1). Both IGL and BX1 enzymes evolved from TSA but independently in monocots and eudicots^25,26^. Unlike TSA, they do not require allosteric activation by TSB to efficiently produce indole^17^. IGL is active as a monomeric enzyme and has been reported to produce indole for volatile emission^27^ and specialized metabolite biosynthesis^2^ in different species of the Poaceae^27,28^, a family of monocots, and in basal eudicots^2^ (Fig. 1a). BX1, in contrast, acts as a homodimer and produces indole for the biosynthesis of BXD^1,29,30^. BX1 enzymes have been exclusively found in BXD-producing plants belonging to the Poaceae^1,25,31^ and in *Consolida orientalis*^26^, a species belonging to the plant family Ranunculaceae (basal eudicots) (Fig.1a). In general, IGP lyases including TSA, IGL and BX1 are localized in the chloroplast^32^, the site of indole and tryptophan biosynthesis^33,34^. However, two cytosolic IGP lyases, named TSA-like and indole synthase (INS), have been reported in the Poaceae and in the Brassicaceae, respectively^17,35^. While maize (*Zea mays*) TSA-like did not display indole biosynthetic activity either alone or in complex with TSB^17^, *Arabidopsis thaliana* INS possesses IGP lyase activity and is likely involved in tryptophan-independent auxin biosynthesis^35,36^.

Although indole emission has been reported in numerous species of the core eudicots^6,8–10,12,15,37^ and many core eudicots use indole as a precursor for BXD biosynthesis^26,38,39^, IGL and BX1 enzymes have not been identified in this taxonomic group. Therefore, it remained unclear how these plants produce indole for volatile emission and specialized metabolite biosynthesis. Recently, the genes responsible for BXD biosynthesis were identified in two core eudicot species, *Lamium galeobdolon* (Lamiaceae) and *Aphelandra squarrosa* (Acanthaceae)^26,40^. However, the first committed step of the pathway, indole formation (Fig. 1b and Supplementary Fig. 1), remained in part elusive. The IGP lyases of these two species, *Lg*IGL1 (EU747715), *Lg*IGL2 (EU747716) and *As*IGL (EU747710), were reported to have low indole biosynthetic activity in vitro compared to the BX1 enzymes from *Z.* *mays* (AY254104) and *C.* *orientalis* (EU747712); therefore, it was unclear whether they provide indole for BXD biosynthesis in planta^26^. In this work, we report the identification of a core eudicot-specific TSB-like pseudoenzyme that itself lacks tryptophan biosynthetic activity but allosterically activates TSA for efficient production of indole for BXD biosynthesis and, more broadly, for plant defense and communication.

## Results

### TSB-like enables indole biosynthesis in core eudicots

**Fig. 2:**
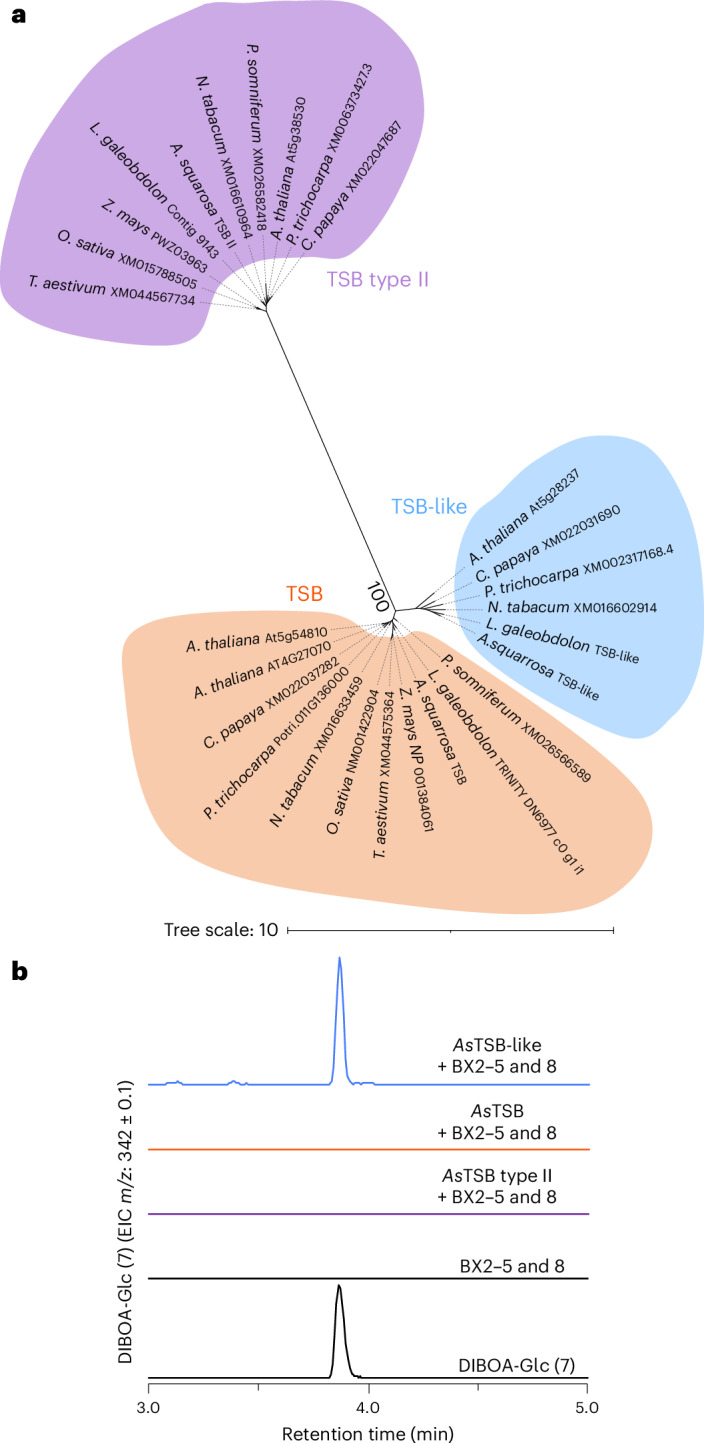
Phylogenetic analysis and indole biosynthetic activity of TSB, TSB-like and TSB type II enzymes. **a**, TSB, TSB-like and TSB type II enzymes form distinct phylogenetic clades. A maximum-likelihood tree was inferred using amino acid sequences. **b**, Transient expression of *AsTSB-like*, *AsTSB* and *AsTSB type II* in *N.* *benthamiana* along with maize *Bx2*, *Bx3*, *Bx4*, *Bx5* and *Bx8* genes. EIC, extracted ion chromatogram. The chromatogram scale (*y* axis, peak intensity) is equalized across all chromatograms.

Phylogenetic analysis showed that the previously identified enzymes *Lg*IGL1, *Lg*IGL2 and *As*IGL clustered with previously characterized TSA enzymes from other core eudicot species and not with IGL or BX1 enzymes (Supplementary Fig. 2), suggesting a function as TSA rather than IGL or BX1. We, therefore, renamed *Lg*IGL1 and *Lg*IGL2 from *L.* *galeobdolon* and *As*IGL from *A.* *squarrosa* as TSA (*Lg*TSA1, *Lg*TSA2 and *As*TSA, respectively). To test whether *Lg*TSA1, *Lg*TSA2 and *As*TSA could nevertheless provide indole for BXD formation in planta, we first reconstituted the BXD biosynthetic pathway in the heterologous host *Nicotiana benthamiana* by transient expression of the BXD biosynthetic genes *Bx1*, *Bx2*, *Bx3*, *Bx4*, *Bx5* and *Bx8* from *Z.* *mays* and then replaced *Bx1* with *LgTSA1*, *LgTSA2* or *AsTSA*. As expected, plants expressing *Bx1*, *Bx2*, *Bx3*, *Bx4*, *Bx5* and *Bx8* accumulated substantial amounts of BXD while plants in which *Bx1* was replaced by *LgTSA1*, *LgTSA2* or *AsTSA* showed significantly lower BXD accumulation (Fig. 1c), indicating that *Lg*TSA1, *Lg*TSA2 and *As*TSA did not function as BX1 or IGL. We, therefore, hypothesized that *A.* *squarrosa* and *L.* *galeobdolon* must have an alternative mechanism to produce indole for BXD biosynthesis. To uncover this mechanism, we screened transcriptomes from *A.* *squarrosa* and *L.* *galeobdolon* for genes coexpressed with *Bx* genes previously identified in these species^40^. This approach revealed in both species a gene that was similar (56% nucleotide sequence identity on average) to *TSB*, named *AsTSB-like* and *LgTSB-like*, which exhibited a Pearson correlation coefficient of 0.96 and 0.99 with *Bx4* and *Bx5* genes in *A.* *squarrosa* and *L.* *galeobdolon*, respectively (Supplementary Figs. 3 and 4). Transient expression of *AsTSB-like* or *LgTSB-like* together with maize *Bx2*, *Bx3*, *Bx4*, *Bx5* and *Bx8* in *N.* *benthamiana* resulted in BXD levels comparable to those produced by plants expressing the entire maize BXD pathway (Fig. 1c). In addition, *N.* *benthamiana* plants expressing only *AsTSB-like* accumulated substantial amounts of indole, whereas indole was barely detectable in plants overexpressing *AsTSA* (Fig. 1d). A comprehensive basic local alignment search tool (BLAST) analysis revealed that TSB-like sequences are not only present in the BXD-producing species *A.* *squarrosa* and *L.* *galeobdolon* but also widespread in all core eudicot species examined (Fig. 1a and Supplementary Fig. 5). Notably, no *TSB-like* gene was found in monocots and basal eudicots (Supplementary Fig. 5), which are known to possess functional IGL and BX1 enzymes for indole production (Fig. 1a and Supplementary Fig. 2)^26,27,41^. Testing of additional *TSB-like* genes from non-BXD-producing species of the core eudicots, including *N.* *benthamiana*, *Populus trichocarpa* and *A.* *thaliana*, showed that all, when transiently overexpressed with maize *Bx2*, *Bx3*, *Bx4*, *Bx5* and *Bx8* in *N.* *benthamiana*, promoted indole formation, as inferred from the production of BXD (Fig. 1c). Phylogenetic analysis showed that TSB-like sequences formed a clade well separated from those of TSB and TSB type II, which are both known to catalyze the formation of tryptophan (Fig. 2a)^42,43^. In contrast to *AsTSB-like*, transient overexpression of *AsTSB* or *AsTSB type II* with maize *Bx2*, *Bx3*, *Bx4*, *Bx5* and *Bx8* in *N.* *benthamiana* did not result in the formation of BXD (Fig. 2b).

### TSB-like is a pseudoenzyme that allosterically activates TSA

**Fig. 3:**
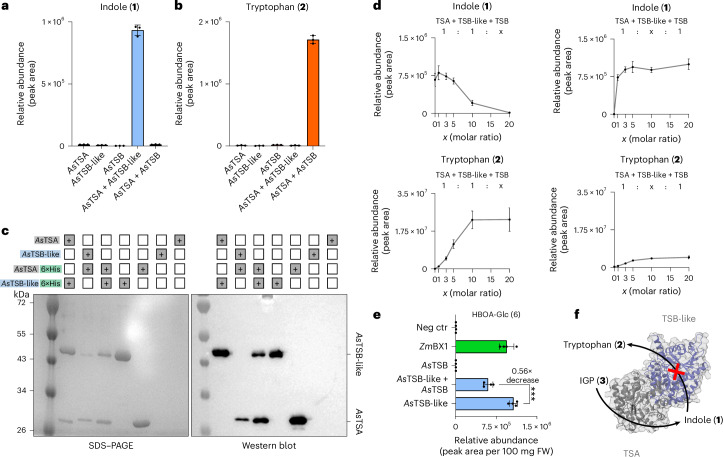
TSB-like binds to TSA and triggers its allosteric activation, resulting in indole formation. **a**,**b**, *As*TSB-like promotes indole formation by TSA but does not produce tryptophan in vitro. Recombinant proteins were assayed with the substrates IGP and l-serine. Accumulation of indole (**a**) and tryptophan (**b**) was measured by LC–MS/MS. Bar graphs represent the mean ± s.d. for three technical replicates (*n* = 3, assays). **c**, TSB-like forms a complex with TSA. Copurification of C-terminal His-tagged and untagged *As*TSA and *As*TSB-like through affinity purification. Untagged TSA or TSB-like could be copurified with the corresponding tagged partner as shown in SDS–PAGE and western blot. **d**, *As*TSB and *As*TSB-like compete for *As*TSA in vitro. Recombinant proteins were assayed with the substrates IGP and l-serine. Left: equimolar concentrations of TSA and TSB-like were incubated with increasing concentrations of TSB, resulting in increased accumulation of tryptophan and reduced accumulation of indole. Right: equimolar concentrations of TSA and TSB were incubated with increasing concentrations of TSB-like, resulting in increased accumulation of indole and tryptophan. The *x* axis shows the molar ratio of the indicated protein. Points represent the mean ± s.d. for three technical replicates (*n* = 3 assays). **e**, *As*TSB and *As*TSB-like compete for TSA in *N.* *benthamiana*. Coexpression of *Z. mays Bx2*, *Bx3*, *Bx4* and *Bx8* with *AsTSB* and *AsTSB-like* compared to *AsTSB-like* resulted in a ~50% decrease in HBOA-Glc produced. Bar graphs represent the mean ± s.d. for four independent biological replicates (*n* = 4 plants). ***P < 0.0005 (two-tailed *t*-test; *P* = 0.0002, *t* = 8.219, df = 6, 95% confidence interval (CI) = −608,573, −329,332). **f**, Schematic of how TSB-like and TSA produce indole (**1**). For visual clarity, only one αβ-like dimer is shown. Source data

TSA and canonical TSB form a protein complex, thereby mutually activating each other. Thus, we hypothesized that indole resulting from the expression of TSB-like might be associated with TSA. In this model, the indole formation observed in *N.* *benthamiana* expressing *TSB-like* genes from other species (Fig. 1c,d and Supplementary Fig. 6) would be because of the interaction between the introduced TSB-like and the endogenous TSA of *N.* *benthamiana*, which is constitutively expressed in the leaves of this plant. Indeed, when recombinant *As*TSB-like was incubated with *As*TSA and the substrates IGP and l-serine, indole but not tryptophan was produced. Conversely, in reactions containing *As*TSA and canonical *As*TSB, tryptophan was the main product. Each enzyme showed negligible activity when assayed alone (Fig. 3a,b), indicating that *As*TSB-like acts as allosteric activator for *As*TSA. Allosteric activation of TSA by TSB-like was also observed upon in vitro coincubation of TSA and TSB-like from several species, including *A.* *thaliana*, *N.* *benthamiana*, *P.* *trichocarpa* and *L.* *galeobdolon* (Supplementary Fig. 7a). In species harboring two TSA genes (for example, *L.* *galeobdolon*)^26^, allosteric activation of both TSA homologs by TSB-like was observed (Supplementary Fig. 7a). Moreover, INS from *A.* *thaliana* displayed allosteric activation upon incubation with *A.* *thaliana* TSB-like (Supplementary Fig. 7a). To test whether TSB-like and TSA form a protein complex that is characteristic of this allosteric activation, we performed copurification assays with different combinations of His-tagged or nontagged recombinant proteins. Incubation of His-tagged *As*TSB-like with untagged *As*TSA or vice versa, followed by nickel affinity purification, always resulted in purification of both proteins, regardless of whether the His tag was fused to the C or N terminus of the protein (Fig. 3c and Supplementary Fig. 7b,c). This demonstrated that *As*TSB-like forms a complex with *As*TSA that is stable even under the conditions of the in vitro purification procedure. Indeed, a comparison of the amino acid residues that form the interface between plant TSA and TSB^44,45^ or TSB-like showed that 69% of the residues were conserved between TSB and TSB-like (Supplementary Figs. 8 and 9). Competition assays in vitro and in *N.* *benthamiana* also suggested that TSB-like and TSA form a complex. In vitro, incubation of TSA and TSB-like with increasing amounts of TSB resulted in a progressive reduction in indole and increase in tryptophan accumulation (Fig. 3d). Incubation of TSA and TSB with increasing amounts of TSB-like resulted in increased indole accumulation and, in this case, minor levels of tryptophan (Fig. 3d). The low levels of tryptophan observed in this experiment were most likely because of the previously reported ability of TSB and the TSA–TSB complex to use indole as a substrate for tryptophan biosynthesis^17,43,46^ (Supplementary Fig. 10). In *N.* *benthamiana*, where the endogenous *TSA* is constitutively expressed for tryptophan biosynthesis, coexpression of *AsTSB-like* together with *AsTSB* and maize *Bx2*, *Bx3*, *Bx4* and *Bx8* resulted in an approximately 50% reduction in the amount of BXD produced compared to the control, which expressed only *AsTSB-like* and maize *Bx2*, *Bx3*, *Bx4* and *Bx8* (Fig. 3e). Moreover, the subcellular localization of *As*TSA and *As*TSB-like, as evidenced by expression of fluorescence-tagged proteins in *N.* *benthamiana*, indicated that both proteins colocalized in the chloroplasts (Fig. 1e and Supplementary Fig. 11). Taken together, our results suggest that TSB-like has no enzymatic function but instead binds to TSA, thereby activating this enzyme for efficient indole production (Fig.3f).

### Two residues mediate the functionalities of TSB-like

**Fig. 4:**
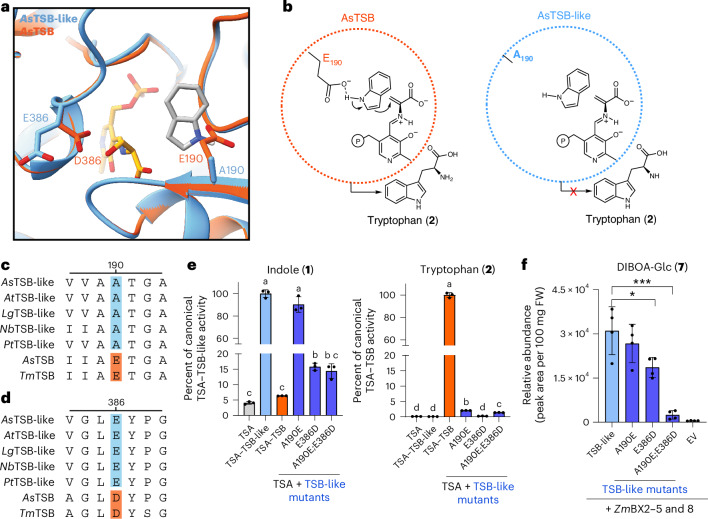
A190 and E386 have a role in *As*TSB-like activity. **a**, Model of the *As*TSB-like and *As*TSB active site showing A190 and E386. The structure of *As*TSB-like was modeled on the crystal structure of *Salmonella* Typhimurium TSB (Protein Data Bank 7JMQ) in open conformation with PLP (yellow). Indole (gray) was docked in silico. **b**, Schematic depiction of the role of E190 in tryptophan biosynthesis. **c**,**d**, Alignment displaying the conservation of A190 and E386. TSB-like enzymes from *A.* *squarrosa*, *A.* *thaliana*, *L.* *galeobdolon*, *N.* *benthamiana* and *P.* *trichocarpa* were compared to *A.* *squarrosa* and *T.* *maritima* TSB. Sequences were aligned using the MUSCLE algorithm and the residues of interest are highlighted in blue for TSB-like and orange for TSB. **e**, *As*TSB-like mutants resulted in a gain of tryptophan biosynthetic activity and reduction in indole biosynthetic activity. Recombinant proteins were assayed on IGP and l-serine. Reaction products were analyzed using LC–MS/MS. Bar graphs represent the mean ± s.d. for three technical replicates (*n* = 3 assays). Columns labeled with different letters represent statistically significant differences (*P* < 0.05, Brown–Forsythe and Welch ANOVA with Dunnett test for multiple comparisons). **f**, The double mutant *As*TSB-like-A190E;E386D showed highly reduced indole production in *N.* *benthamiana*, as evidenced by monitoring production of the BXD DIBOA-Glc. *As*TSB-like mutants were transiently expressed in *N.* *benthamiana* along with *Z.* *mays Bx2*, *Bx3*, *Bx4*, *Bx5* and *Bx8*. Bar graphs represent the mean ± s.d. for four independent biological replicates (*n* = 4 plants). ***P < 0.0005 (two-tailed *t*-test; *P* = 0.0004, *t* = 6.946, df = 6, 95% CI = −38,675, −18,525) and *P < 0.05 (two-tailed *t*-test; *P* = 0.03, *t* = 2.830, df = 6, 95% CI = −23,181, −1,683). Source data

Phylogenetic analysis suggested that *TSB-like* most likely evolved by gene duplication and neofunctionalization of a canonical *TSB* gene (Supplementary Fig. 5). To understand how TSB-like lost tryptophan synthase activity but retained the capacity to allosterically activate TSA, we identified residues that were consistently different between TSB and TSB-like in all species examined (Supplementary Fig. 12). One of the identified residues, E190, numbered according to *As*TSB (Supplementary Fig. 13) and corresponding to E105 in the model TSB of *Thermotoga maritima*, is located in the active site (Fig. 4a) and has been shown to be essential for tryptophan formation^21,22,46^. This residue activates indole by coordinating the N–H proton through its side-chain carboxyl group^46^ (Fig. 4b). In TSB-like, this glutamate residue is almost always replaced by alanine (Fig. 4c and Supplementary Fig. 14), with the only exception found in TSB-like from Solanaceae species, which instead contained a serine or a proline at this position (Supplementary Fig. 15). Site-directed mutagenesis of A190 in *As*TSB-like to glutamate resulted in a gain of tryptophan biosynthetic activity, albeit at low levels (Fig. 4e). Structure modeling indicated that the E190A substitution does not cause major alterations of the active site structure, suggesting that the activation effect of glutamate is only because of the carboxyl group of the side chain (Fig. 4b and Supplementary Fig. 16). Nevertheless, the *As*TSA–*As*TSB-like-A190E complex still produced indole in amounts comparable to those of *As*TSA–*As*TSB-like (Fig. 4e and Supplementary Fig. 17b). Another residue that was consistently different between TSB and TSB-like, D386, which corresponds to D300 in the TSB from *T.* *maritima*, has been proposed to have a role in TSB activation^21,47^. Binding of IGP to TSA triggers a conformational change in TSA that promotes a switch from the inactive to the active conformation of TSB. By forming a salt bridge with R222, D386 stabilizes the active TSB conformation^21^, which in turn promotes IGP cleavage and indole biosynthesis by TSA. This aspartate residue was universally replaced by glutamate in TSB-like (Fig. 4a,d and Supplementary Fig. 14). Site-directed mutagenesis of E386 in *As*TSB-like to aspartate resulted in reduced indole formation, although tryptophan biosynthesis was still not observed (Fig. 4e and Supplementary Fig. 17a). We hypothesize that the glutamate residue in TSB-like sequences, in contrast to the shorter aspartate in TSB, always interacts with the positively charged residue at position 222 (arginine or lysine in TSB-like sequences), regardless of the TSA conformation, resulting in permanent stabilization of the active conformation of TSB-like (Supplementary Figs. 18 and 19). The double mutant *As*TSB-like-A190E;E386D, together with *As*TSA, combined the gain of tryptophan biosynthetic activity with a reduction in indole biosynthetic activity (Fig. 4e and Supplementary Fig. 17). Moreover, transient expression of the double mutant in *N.* *benthamiana* with maize *Bx2*, *Bx3*, *Bx4*, *Bx5* and *Bx8* showed that TSB-like activity was almost completely eliminated in planta, as evidenced by the low levels of BXD production (Fig. 4f). Introducing the reverse substitutions into *As*TSB (*As*TSB-E190A and *As*TSB-E190A;D386E) resulted in the loss of tryptophan biosynthetic activity but not in a gain of indole biosynthetic activity (Supplementary Fig. 20). Site-directed mutagenesis of additional residues, which differed between TSB and TSB-like, was not sufficient to increase indole production (Supplementary Fig. 21). Interestingly, it was recently reported that site-directed mutagenesis of E105 to glycine, alanine and serine enabled bacterial TSB enzymes to use phenolic substrate to produce tyrosine (**4**) and tyrosine analogs^48^. However, testing *As*TSB-like enzymes on phenol (**5**) showed no tyrosine biosynthetic activity (Supplementary Fig. 22).

The data presented here indicate that the substitution of E190 to alanine and D386 to glutamate had a notable role in the evolution of TSB-like from TSB, resulting in the loss of tryptophan biosynthetic activity and conservation of TSA activation. However, the lower tryptophan biosynthetic activity exhibited by TSB-like-A190E;E386D compared to TSB suggests that a more extensive network of residues may regulate efficient tryptophan biosynthesis.

### TSB-like is involved in plant defense and signaling

**Fig. 5:**
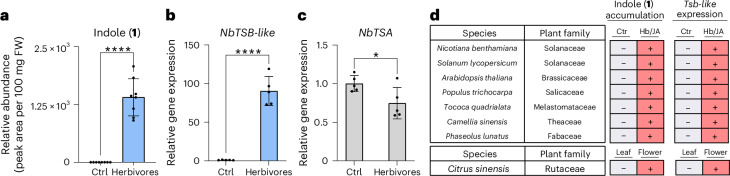
Indole accumulation and emission are accompanied by *TSB-like* expression in core eudicots. **a**, *N.* *benthamiana* plants wounded by *S.* *littoralis* caterpillars accumulated indole. Plants were exposed to herbivory for 17 h. Bar graphs represent the mean ± s.d. for eight independent biological replicates (*n* = 8 plants). ****P < 0.0001 (two-tailed *t*-test; *P* = 0.0000001, *t* = 9.865, df = 14, 95% CI = −1,710, −1,100). **b**,**c**, Relative gene expression of *TSB-like* and *TSA* in *N.* *benthamiana* plants treated with *S.* *littoralis* caterpillars. Bar graphs represent the mean ± s.d. for five independent biological replicates (*n* = 5 plants). ****P < 0.0001 (two-tailed *t*-test; *P* = 0.00000574, *t* = 10.54, df = 8, 95% CI = 69.80, 108.9) and *P < 0.05 (two-tailed *t*-test; *P* = 0.04, *t* = 2.486, df = 8, 95% CI = −0.4896, −0.01843). **d**, Meta-analysis showing the accumulation or emission of indole and the expression of *TSB-like* in different families of core eudicots upon herbivory (Hb) or jasmonic acid (JA) treatment. (+) indicates increased accumulation or emission of indole or increased expression of *TSB-like* compared to the control. Source data

Indole is a widespread plant volatile that is often released in response to herbivore damage or as a characteristic floral scent component. *N.* *benthamiana* has been reported to emit indole upon herbivore attack^8^ (Fig. 5a). We could show that, along with indole emission, *TSB-like* expression but not *TSA* expression was strongly upregulated in *N.* *benthamiana* upon herbivore damage (Fig. 5b,c), suggesting that TSB-like promotes indole formation in response to biotic stress. A meta-analysis of literature data across core eudicot species for which both metabolomic and transcriptomic data were available revealed that both herbivory-induced and floral-scent-related indole emissions were always accompanied by an upregulation of *TSB-like* expression, whereas *TSA* expression remained unchanged or showed smaller fold-change differences compared to TSB-like (Fig. 5d and Supplementary Fig. 23). These observations are consistent with a recently reported study from tea that showed the upregulation of a TSB-like protein after herbivore attack and that this protein interacts with TSA^11^. Along with the absence of *IGL* and *BX1* genes, these data suggest that indole emission in core eudicots is dependent on the action of TSB-like.

## Discussion

Despite the biological importance of indole in plant defense and communication, the mechanism underlying its formation in the vast clade of the core eudicots remained unknown. In this work, we report that the pseudoenzyme TSB-like, a catalytically ‘dead’ paralog of TSB, appears to be responsible for indole biosynthesis in core eudicots. TSB-like most likely evolved from TSB through a loss of tryptophan biosynthetic activity. The resulting catalytically inactive TSB-like mimics the interaction of TSB with TSA, thereby allosterically activating TSA to allow indole biosynthesis but without subsequent conversion to tryptophan (Fig. 3f). Therefore, TSB-like has evolved to serve as a switch that toggles between tryptophan and indole biosynthesis by hijacking the pre-existing TSA. However, tryptophan is an essential amino acid and plants must be able to maintain tryptophan biosynthesis even under conditions where indole is produced for defense or communication. It is, therefore, conceivable that TSB-like and TSB, which both compete for TSA, may be expressed in different cell types, especially in plants where TSB-like is continuously expressed (for example, for BXD production, as in *L.* *galeobdolon* and *A.* *squarrosa*). Single-cell techniques could be used in future studies to understand how core eudicots control TSA-dependent indole formation for tryptophan biosynthesis, defense and communication. Although pseudoenzymes can be challenging to discover, recent work has highlighted that these proteins have essential roles in a number of biological processes in plants such as vitamin B_6_ biosynthesis^49^, alkaloid biosynthesis^50^ and starch breakdown^51,52^. In summary, we report the biosynthesis of indole, a fundamental part of the plant defense response^53^, in core eudicots. The elucidation of TSB-like, therefore, paves the way for the metabolic engineering of indole biosynthesis for plant defense, pollinator attraction and specialized metabolite biosynthesis in core eudicots.

## Methods

### Chemicals

All chemicals used in this study were purchased at molecular biology grade or higher from Sigma-Aldrich, Thermo Fisher or Tokyo Chemical Industry unless otherwise stated. BXD standards previously synthesized or isolated^40^ were used in this study.

### Plant material and growth

*A.* *squarrosa*, *L.* *galeobdolon* and *N.* *benthamiana* plants were cultivated under greenhouse conditions as previously described^40^. *N.* *benthamiana* plants were grown for 3 weeks before gene candidate infiltration.

### Plant metabolite extraction

Collected plant material was snap-frozen in liquid nitrogen and ground to fine powder with 3-mm tungsten carbide beads using a TissueLyser (Qiagen) or, when more material was needed, liquid-nitrogen-frozen samples were ground to a fine powder in a prechilled mortar. Tissue samples (100 mg ± 5%) were extracted with 500 μl of methanol (liquid chromatography–mass spectrometry (LC–MS) grade). Samples were vortexed vigorously and then incubated at 25 °C, shaking for 15 min, followed by centrifugation at maximum speed before filtering with a 0.22-μm PTFE syringe filter for LC–MS analysis.

### Gene candidate identification

Gene candidates were selected from the previously published *A.* *squarrosa* and *L.* *galeobdolon* transcriptomes (BioProject PRJNA967136) assembled as previously reported^40^. Pearson coexpression correlation analyses were performed in Excel. TSB-like candidates in other species were identified on the basis of homology by performing BLAST analyses on public databases: National Center for Biotechnology Information (NCBI), SolGenomics, Citrus Genome Database, 1KP and NbenBase.

### Cloning

Total RNA was extracted from ground plant tissue using the RNeasy plant mini kit (Qiagen) including an on-column DNAse digestion step and complementary DNA (cDNA) was synthesized from total RNA using SuperSCript IV VILO Master Mix (Thermo Fisher Scientific), according to the manufacturer’s instructions. Genes were amplified from cDNA using Platinum SuperFi II PCR master mix (Thermo Fisher Scientific). Synthetic genes, when used, were ordered from Twist Bioscience and used as a template for PCR amplification. PCR products were purified using DNA clean and concentrator 5 (Zymo) or Zymoclean gel DNA recovery kit (Zymo). Amplified genes were inserted with In-Phusion HD cloning (Takara Bio) in p3Ω1 vector (BsaI-HF digested) for expression in *N.* *benthamiana*. For expression in *Escherichia coli*, the following vectors were used: pOPINF (HindIII-HF and KpnI-HF digested) for N-terminal His-tagged sequences, pOPINE (NcoI-HF and KpnI-HF digested) for C-terminal His-tagged sequences and pET28a for alternative N-terminal (BamHI-HF and NotI-HF digested) or C-terminal (NcoI-HF and XhoI-HF digested) His tagging. For subcellular localization studies, *AsTSA* and *AsTSB-like* were cloned with a C-terminal fused fluorescent protein (mCeruleans and eYFP) separated by an AGCGGC linker. The fusion constructs were cloned under the control of the strong constitutive *Solanum lycopersicum* Ubiquitin 10 (*Sl*Ubq10) promoter and terminator in 3α1 vector through Golden Braid using BsaI-HF and T4 DNA^55^. Vectors harboring the sequences of interest were transformed in *E.* *coli* Top10 with the heat-shock method. Plasmid DNA was isolated using Wizard Plus SV Minipreps DNA purification system kit (Promega) following the manufacturer’s instructions. Each construct was checked through Sanger sequencing. All primers used in this study are reported in Supplementary Table 2.

### Transient transformation of *N.benthamiana*

Electrocompetent *Agrobacterium tumefaciens* GV3101 (Goldbio) cells were mixed with sequence-confirmed plasmid and incubated on ice for 15 min. Cells were electroporated using a BioRad Micropulser. The transformed cells were recovered in 1 ml of Luria–Bertani (LB) medium and incubated at 28 °C, 200 rpm for 3 h before plating on LB–agar plates containing the appropriate selection marker. Plates were incubated at 28 °C for 48 h. Single colonies were inoculated into liquid LB medium with the appropriate selection and incubated overnight at 28 °C, 200 rpm. For *N.* *benthamiana* transient transformation, the overnight cultures were pelleted by centrifugation at 3,220*g* for 10 min at 14 °C. The cell pellet was resuspended in infiltration medium (10 mM MES, 10 mM MgCl_2_ and 100 µM acetosyringone, pH 5.7) to an optical density at 600 nm (OD_600_) of 0.6–0.7 and incubated at 28 °C, 200 rpm for 1.5 h. Equal volumes of the prepared infiltration solutions were mixed to achieve the desired transformation mix containing each construct at an OD_600_ of 0.1. The transformation mix was infiltrated into the abaxial side of 3-week-old *N.* *benthamiana* leaves using a needleless 1-ml syringe. The infiltrated plants were maintained in a growth chamber under growth lights up to 5 days after infiltration, when samples were collected. In all transformations, a construct encoding the silencing repressor protein p19 was coinfiltrated to enhance expression.

### Small-scale expression of candidate genes in *E.coli*

Gene candidates were expressed as previously described^40^ with minor modifications. In brief, *E.* *coli* DE3 (Thermo Fisher Scientific) cells were transformed with sequence-confirmed plasmids using the heat-shock method. Single colonies were inoculated in liquid LB medium with selection and grown at 37 °C, 250 rpm, overnight. The seed culture (1 ml) was used to inoculate 100 ml of 2× YT medium with selection and the culture was grown at 37 °C, 250 rpm, until OD_600_ = 0.5–0.6. Cultures were then incubated at 18 °C, 250 rpm, for 20 min before the addition of 500 µM IPTG. Induced cultures were incubated at 18 °C, 250 rpm, overnight. Cultures were retrieved by centrifugation (4,000*g*, 4 °C, 15 min) and resuspended in A1 buffer (50 mM Tris-HCl, 50 mM glycine, 5% v/v glycerol, 0.5 M NaCl and 20 mM imidazole, pH 8) with 0.2 g L^−1^ lysozyme, one tablet (50 ml) of EDTA-free protease inhibitor (and 100 μM PLP for TSB and TSB-like) and disrupted by sonication on ice (Bandelin UW 2070). Cell debris was removed by centrifugation at 35,000*g* at 4 °C for 20 min and His-tagged proteins were purified from the supernatant using Ni-NTA agarose (Qiagen) beads according to the manufacturer’s instructions. Proteins were eluted using elution buffer B1 (A1 buffer + 500 mM imidazole, pH 8). Ultimately, elution buffer was exchanged for protein storage buffer (20 mM HEPES and 150 mM NaCl, pH 7.5, with 10% glycerol) using Amicon concentrator columns (Merck Millipore). Proteins were aliquoted and stored at −20 °C.

### Large-scale expression of candidate genes in *E.coli*

For large-scale heterologous expression, 1 L of 2× YT medium was inoculated with 10 ml of seed culture and induced as described above. Pelleted cells were resuspended in 20 ml of A1 buffer with 0.2 g L^−1^ lysozyme, one tablet (50 ml) of EDTA-free protease inhibitor and 100 μM PLP. Cells were disrupted by sonication on ice (Bandelin UW 2070). Cell debris was removed by centrifugation at 35,000*g* at 4 °C for 20 min and His-tagged proteins were purified on an ӒKTA pure fast protein LC system (GE Healthcare) equipped with a 5-ml HisTrap column (Cytiva). The fast protein LC system was programmed as previously described^56^. In brief, the column was equilibrated with five column volumes of buffer A1. The protein sample was loaded at a flow rate of 2 ml min^−1^. Subsequently the column was washed with buffer A1 (flow rate of 5 ml min^−1^) for a total of ten column volumes. The protein was eluted with five column volumes of buffer B1 and the elution monitored using ultraviolet absorption at 280 nm.

### Protein concentration determination

The concentration of PLP-dependent protein was calculated using the Pierce Rapid Gold BCA protein assay kit (Thermo Fisher Scientific) following the manufacturer’s instructions. Plates were read on a CLARIOstar Plus (BMG Labtech) plate reader. The concentration of non-PLP-dependent proteins was determined spectrophotometrically measuring absorbance at 280 nm on an IMPLEN Nanodrop.

### SDS–PAGE and Western blot

SDS–PAGE analyses were performed using Novex 12%, Tris–glycine Plus WedgeWell gels (Invitrogen) according to the manufacturer’s instructions. Gels for SDS–PAGE were stained with Quick Coomassie stain (Serva). Gels for western blot analysis were transferred on a Power Blotter Select Transfer Stack PVDF mini size membrane using Power Blotter XL transfer station (Invitrogen). Blotted membranes were blocked in TBS + 1 ml L^−1^ Tween buffer (TBST) + 5% (w/v) skimmed milk at room temperature for 1 h. Blocking solution was removed and membranes were incubated in TBST + 3% (w/v) skimmed milk and anti-His antibody coupled with horseradish peroxidase (BioRad, MCA5995P; 1:1,000) as per the manufacturer’s instructions. Antibodies were validated according to ISO 9001: 2015 by the manufacturer as stated on the BioRad website. Western blots were imaged with Clarity Western enhanced chemiluminescence substrate (BioRad) as per the manufacturer’s instructions.

### IGP in vitro biosynthesis

IGP was synthesized in vitro as described by Ivens et al.^57^ by incubating recombinantly purified *E.* *coli* phosphoribosyl transferase (*TrpD*) and phosphoribosyl anthranilate isomerase–IGP synthase (*TrpF*–*TrpC* fusion gene) with 0.5 mM MgCl_2_, 0.4 mM DTT, 3 mM anthranilic acid and 3 mM 5-phospho-d-ribose-diphosphate. The reaction was performed in KPO_4_ buffer, 25 mM (pH 7.5) at 30 °C, shaking for 1 h. The reaction was stopped by heat inactivation at 95 °C for 10 min and proteins were precipitated by centrifugation. IGP was stored at −20 °C and used within 1 day of synthesis.

### In vitro assays

In vitro assays for indole and tryptophan biosynthesis were performed in KPO_4_ buffer, 25 mM (pH 7.5) with 10 nM of each protein and saturating concentrations of IGP, 1 mM l-serine and 0.2 mM PLP. Reactions were started by addition of substrate. The reactions were incubated 15 min at 30 °C, 300 rpm and quenched by the addition of one isovolume of methanol. Proteins were precipitated by centrifugation and samples were analyzed through LC–MS. In vitro reactions to check tyrosine biosynthesis were performed in in KPO_4_ buffer, 25 mM (pH 7.5) with 50 nM of each protein, 1 mM phenol in DMSO, 1.5 mM l-serine and 0.2 mM PLP. Reactions were started by addition of the substrate and incubated for 1 h at 30 °C, 300 rpm. Reactions were quenched by addition of one isovolume of methanol and1 M HCl. Proteins were precipitated by centrifugation and samples were analyzed on LC–quadrupole time-of-flight qTOF) MS.

### LC–qTOF-MS analysis

Samples were analyzed as previously described^40^ with minor variations. LC–qTOF-MS analyses were conducted on a Thermo Scientific UltiMate 3000 ultrahigh-performance LC (UHPLC) system coupled to an Impact II ultrahigh-resolution qTOF-MS instrument (Bruker Daltonics). Chromatographic separation was performed using a reverse-phase Phenomenex Kinetex XB-C18 column (100 × 2.1 mm, 2.6 µm; 100 Å) at 35 °C. The mobile phase consisted of water + 0.1% formic acid (A) and acetonitrile (B) run at a flow rate of 0.3 ml min^−1^ with a sample injection of 2 µl. The chromatographic separation was performed starting at 5% B for 1 min, followed by a linear gradient from 5% to 50% B in 7 min, 100% B for 2.5 min and 5% B for 2.5 min. MS acquisition was performed in positive or negative electrospray ionization (ESI) mode depending on the compound of interest. Data were analyzed using Bruker MS data analysis version 6.1.

### LC–MS/MS analysis

Targeted analysis of indole and tryptophan was performed using a Thermo Scientific UltiMate 3000 UHPLC system coupled to a Bruker EVOQ Elite tandem MS instrument. Chromatographic separation was performed using a reverse-phase Phenomenex Kinetex XB-C18 column (100 × 2.1 mm, 2.6 µm; 100 Å) at 35 °C. The mobile phase consisted of water + 0.1% formic acid (A) and acetonitrile (B) run at a flow rate of 0.3 ml min^−1^ with a sample injection of 1 µl. The chromatographic separation was performed starting at 5% B for 30 s, followed by a linear gradient from 5% to 70% B in 4 min, 100% B for 2 min and 5% B for 2 min. MS acquisition was performed in positive mode using a heated ESI source, with a spray voltage of 4,000 V, cone temperature of 350 °C, cone gas flow of 20 psi, probe temperature of 400 °C, probe gas flow of 45 psi and nebulizer gas flow of 50 psi. Indole and tryptophan were detected using multiple reaction monitoring (MRM) transitions. For indole, the transition from 118 *m/z* to 91 *m/z* using a collision energy of 19 eV was used. For tryptophan, the transitions from 205.1 *m/z* to 188 *m/z* with a collision energy of 5 eV, 205.1 *m/z* to 146 *m/z* with a collision energy of 13 eV and 205.1 *m/z* to 118 *m/z* with a collision energy of 23 eV were used. Data were analyzed using Bruker MS Data Review version 8.2.1 software.

### Confocal laser scanning microscopy

*A.* *tumefaciens* strains harboring *AsTSB-like*–*eYFP* or *AsTSA*–*mCeruleans* constructs were infiltrated in 3-week-old *N.* *benthamiana* plants as described above. Plant leaf disks were analyzed 48 h after infiltration. Micrographs of the freshly punched leaf disks were acquired using a cLSM 880 Axio Imager 2 (Zeiss) equipped with a C-Apochromat ×40/1.20 water immersion objective. The leaf disks were water mounted in 3D-printed object slides with 400-µm-deep circular wells and covered with a 170-µm-thick cover glass. The fluorophores were scanned in two line-sequential tracks with two channels each. The first track contained excitation with a 458-nm argon laser (10% transmission) for mCyan and 405-nm laser diode (1%) for chlorophyll autofluorescence combined with MBS 405 and MBS 458/514. Emissions of mCyan and chlorophyll were detected at 460–499 nm (650 detector gain) and 639–743 nm (650 gain), respectively, with a pinhole adjusted to 1 Airy unit. The line-sequential second track contained excitation of eYFP with a 514-nm Argon laser (3%) combined with MBS 458/514 and its emission was detected at 517–597 nm (600 gain). Additionally, the second track contained a transmitted light channel T-PMT (400 gain). The majority of the micrographs were acquired unidirectionally with an averaging of 8, pixel dwell time of 0.76 µs, resolution of 1,024 × 1,024 and resulting pixel scaling of 50 × 50 nm.

### Herbivory treatment

Three to four *Spodoptera littoralis* caterpillars (second to third instar) were starved for 24 h, placed on 3-week-old *N.* *benthamiana* leaves and left to feed on the plants for 17 h. Afterward, the caterpillars were removed and plant tissue was immediately snap-frozen in liquid nitrogen. Tissue was ground to a fine powder and used for metabolite extraction and qPCR analysis.

### qPCR analysis

Primers for reverse transcription (RT)–qPCR analysis were designed to have a *T*_m_ of 60 °C, a G+C content of 40–60% and a length of 20–21 nt using the primer design software in Geneious Prime (modified Primer3 2.3.7 version), resulting in amplicon sizes between 105 and 134 bp. The specificity of the primers was confirmed by agarose gel electrophoresis, melting curve analysis and sequence verification of the cloned PCR amplicons. The efficiencies of the primers (95.7–103.6%) were determined using a standard curve. Three common housekeeping genes were tested^58^. The most stable gene (*PP2A*) according to the s.d. was used to calculate the relative quantities. All samples were run on a CFX Connect real-time PCR detection system (BioRad) in an optical 96-well plate. RT–qPCR was performed with the Biozym Blue S’Green qPCR kit separate ROX according to the manufacturer’s instructions. cDNA was diluted 1:10 for analysis. Five biological samples per treatment were analyzed in triplicate. The following PCR conditions were applied for all reactions: initial incubation at 95 °C for 3 min followed by 40 cycles of amplification (95 °C for 5 s and 60 °C for 20 s). Reads were taken during the extension step of each cycle and melting curve data were recorded at the end of cycling at 65–95 °C. Normalized fold expression was calculated with the ΔΔCP method^59^. Data and calculations are provided in the Source Data.

### Protein modeling

Protein models were generated using AlphaFold2 in MMSeq (https://colab.research.google.com/github/sokrypton/ColabFold/blob/main/AlphaFold2.ipynb) with default parameters (accession date: 8 March 2024)^60^. Alternatively, models were created by homology modeling using SWISS-MODEL (https://swissmodel.expasy.org/). PLP and ligands were introduced in the models in PyMol by aligning the obtained protein model with crystal structures of orthologous enzymes cocrystallized with PLP and ligands. Protein figures were generated with Chimera X version 1.3.

### Statistics and reproducibility

Statistical analysis was performed using GraphPad Prism version 10.0.3. Statistical tests and parameters used for each experiment are reported in the corresponding figure legend or Source Data. Experiments were performed at least three times with similar results. Confocal microscopy experiments were performed twice with similar results.

### Phylogenetic analysis

Amino acid sequences were aligned with WebPrank alignment software^61^ and maximum-likelihood phylogenetic trees were inferred using iQTree^62^, unless otherwise specified. The phylogenetic tree of plant families was readapted from the Kew Gardens tree of life^54^.

### Reporting summary

Further information on research design is available in the Nature Portfolio Reporting Summary linked to this article.

## Online content

Any methods, additional references, Nature Portfolio reporting summaries, source data, extended data, supplementary information, acknowledgements, peer review information; details of author contributions and competing interests; and statements of data and code availability are available at 10.1038/s41589-025-01943-y.

## Supplementary information

Supplementary Information Supplementary Figs. 1–23, Tables 1 and 2, references and source data for Supplementary Figs. 7b and 17c.

Reporting Summary

Supplementary Data 1 Source data for Supplementary Information.

Supplementary Data 2 Pdb coordinates of the TSA–TSB model protein structure.

## Source data

Source Data Fig. 1 Peak areas.

Source Data Fig. 3 Peak areas.

Source Data Fig. 3 Unprocessed SDS–PAGE and western blot.

Source Data Fig. 4 Peak areas.

Source Data Fig. 5 Peak areas, expression levels, qPCR raw data, metanalysis data and sources.

## Data Availability

Genes described in this study were deposited to NCBI GenBank under the accession numbers given in Supplementary Table 1. Sequences of the previously reported *Lg*IGL1 (EU747715), *Lg*IGL2 (EU747716), *As*IGL (EU747710), *Zm*BX1 (AY254104) and *Co*BX1 (EU747712) were retrieved from NCBI. Other sequences were retrieved from SolGenomics, Citrus Genome Database, 1KP and NbenBase with the accession numbers provided in Supplementary Data 1. *A.* *squarrosa* and *L.* *galeobdolon* transcriptome raw sequence reads used are available from the NCBI Sequence Read Archive under BioProject PRJNA967136. All other data are available in the main text and Supplementary Information. Sequences used for phylogenetic analysis are provided, with accession numbers, in Supplementary Data 1. Other data are available from the corresponding authors upon request. Source data are provided with this paper.
