# Supplementary material for: A pseudoenzyme enables indole biosynthesis in eudicot plants
Source: Nat Chem Biol. 2025 Jun 25;22(1):120–7. doi: 10.1038/s41589-025-01943-y (PMC12727527; doi:10.1038/s41589-025-01943-y)
Supplement: Supplementary file 1 — Supplementary Figs. 1–23, Tables 1 and 2, references and source data for Supplementary Figs. 7b and 17c. [file 41589_2025_1943_MOESM1_ESM.pdf]

nature chemical biology

Supplementary information https://doi.org/10.1038/s41589-025-01943-y

A pseudoenzyme enables indole biosynthesis in eudicot plants

In the format provided by the authors and unedited

Table of Contents

Supplementary Figure 1: BXD biosynthetic pathway from the precursor IGP to DIBOA-Glc.. 3

Supplementary Figure 2: Distribution of IGP-lyases (TSA, IGL, BX1, TSA-like, and INS enzymes) among grasses (monocots) basal eudicots and core eudicots. 4

Supplementary Figure 3: Nucleotide and amino acid sequence identity between TSB-like, TSB, and TSB type II sequences of *Aphelandra squarrosa* (As) and *Lamium galeobdolon* (Lg). 5

Supplementary Figure 4: TSB-like co-express with *Bx* genes in the BXD-producing species *Aphelandra squarrosa* and *Lamium galeobdolon*. 6

Supplementary Figure 5: Amino acid sequences of TSB, TSB-like, and TSB type II form three separate clades. 7

Supplementary Figure 6: Transient expression of *AsTSB-like* and *AsTSA* + *AsTSB-like* in *Nicotiana benthamiana* results in substantial DIBOA-Glc accumulation. 8

Supplementary Figure 7: Testing TSA and TSB-like interaction and allosteric activation. 9

Supplementary Figure 8: Residues forming the TSA-TSB interface. 10

Supplementary Figure 9: TSB residues forming the interface with TSA 11

Supplementary Figure 10: Indole can be used as a substrate both from TSA-TSB complex as well as from TSB alone. 12

Supplementary Figure 11: Subcellular localization of *As*TSA and *As*TSB-like. 13

Supplementary Figure 12: Highly conserved residues that differ between TSB and TSB-like. 14

Supplementary Figure 13: TSB-like reference alignment 15

Supplementary Figure 14: Mutation of two conserved residues in TSB-like and TSB leads to activity changes. 16

Supplementary Figure 15: A190 is not strictly required for TSB-like activity 17

Supplementary Figure 16: Active site of *A. squarrosa* TSB-like (AsTSB-like) and *A. squarrosa* TSB (AsTSB) and effect of site-directed mutagenesis of residue 190 on the active site architecture 18

Supplementary Figure 17: Site directed mutagenesis of two conserved residues in TSB-like and TSB leads to activity changes in vitro 19

Supplementary Figure 18: Active site of *A. squarrosa* TSB-like (AsTSB-like) and *A. squarrosa* TSB (AsTSB) and effect of site-directed mutagenesis at position 386 on the active site architecture 20

Supplementary Figure 19: Orientation of residue 386 and 222 in the inactive and active conformation of *A. squarrosa* TSB-like and TSB 21

Supplementary Figure 20: Site-directed mutagenesis of E190A and D386E is not sufficient to convert *As*TSB into *As*TSB-like 22

Supplementary Figure 21: Site directed mutagenesis of highly conserved residues that differ between TSB and TSB-like clades did not substantially change the activity of the resulting proteins 23

Supplementary Figure 22: *As*TSB-like shows no tyrosine biosynthetic activity 25

Supplementary Figure 23: Expression differences of TSA and TSB-like genes between conditions in which indole emission is induced and the control treatment in different plant species 26

Supplementary Figure 24: Source Data File for Supplementary Figure 7B. 27

Supplementary Figure 25: Source Data File for Supplementary Figure 17C. 28

Supplementary Table 1: Accession and sequences of the genes characterized in this study. 29

Supplementary Table 2: List of primers used in this study. 34

Supplementary References 37

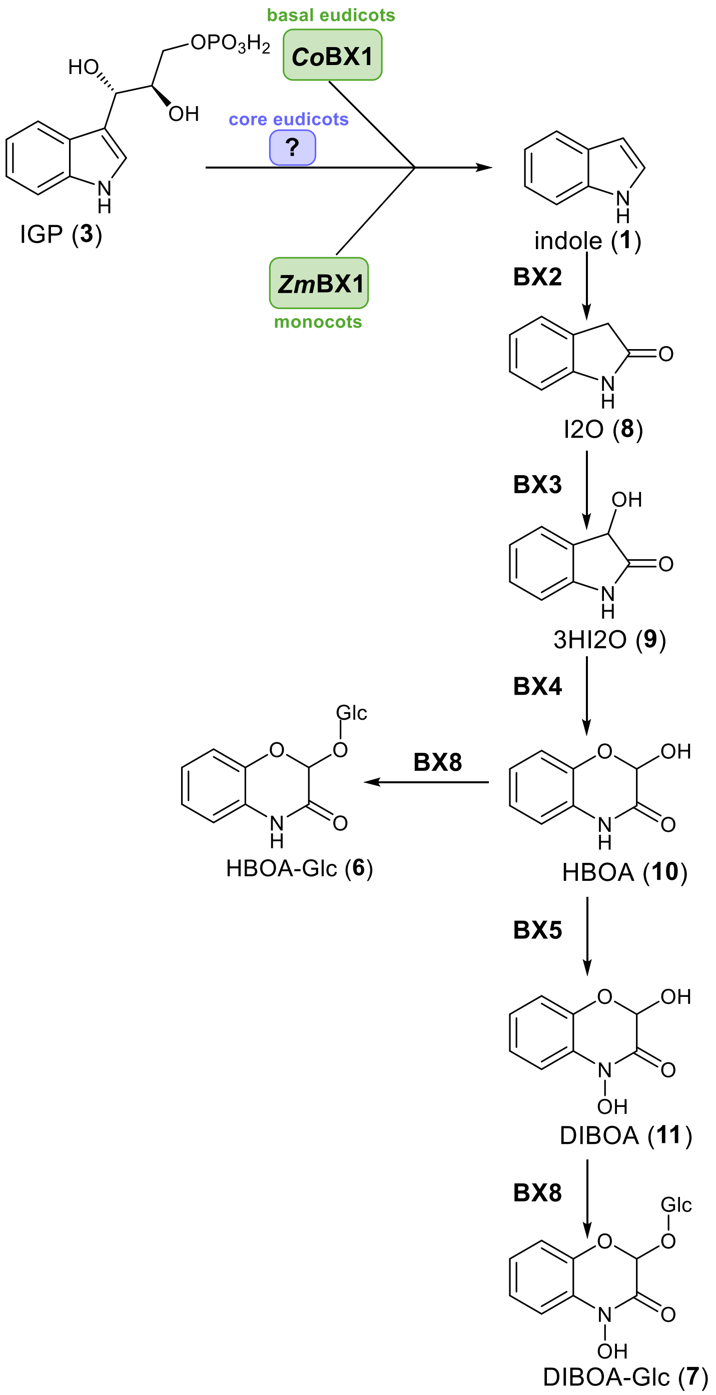

Supplementary Figure 1: BXD biosynthetic pathway from the precursor IGP to DIBOA-Glc. Different indole biosynthetic enzymes produce indole for BXD biosynthesis in monocots, basal eudicots and core eudicots. BX2, BX3, BX4, BX5, and BX8 indicate BXD biosynthetic enzymes.

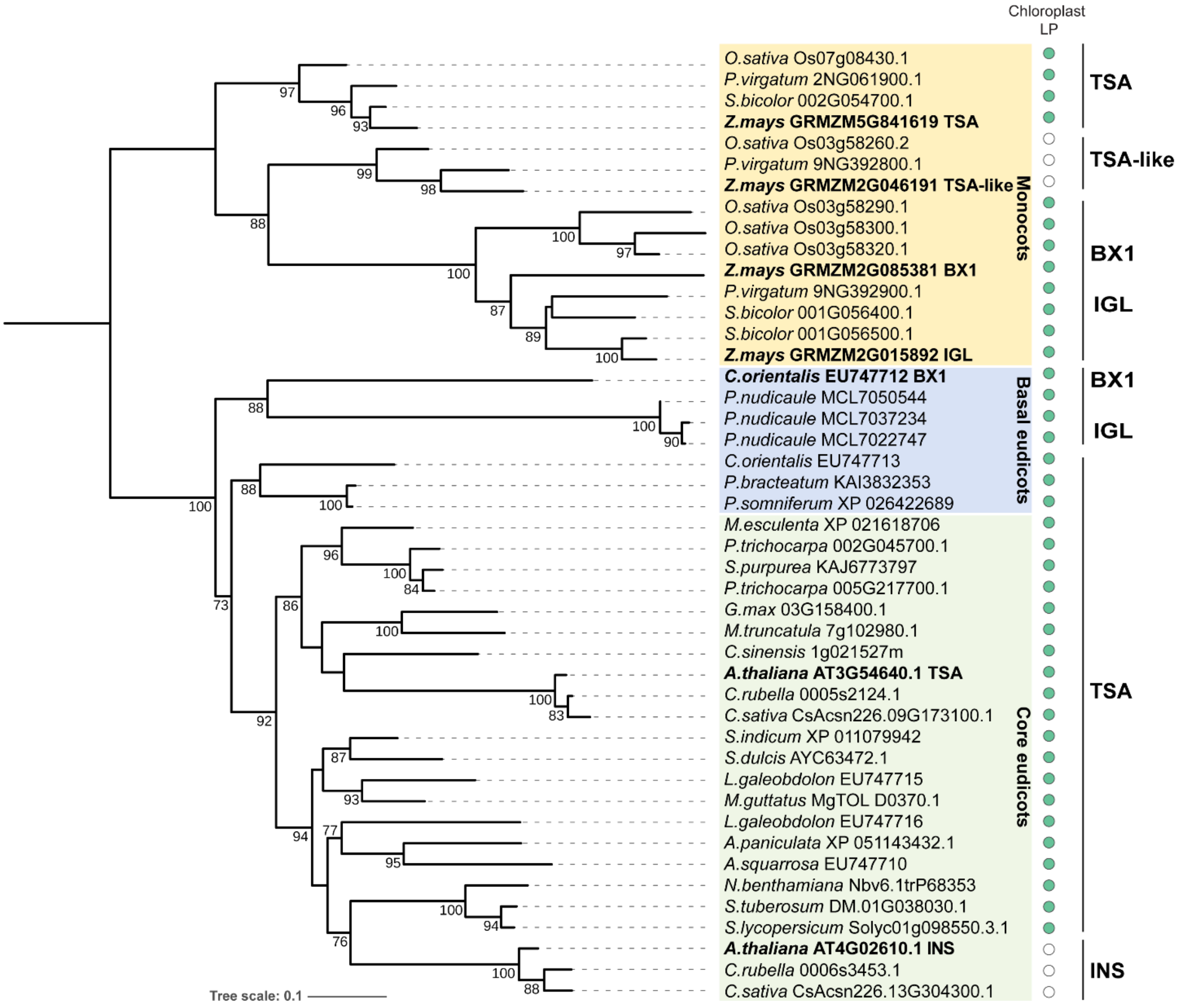

Supplementary Figure 2: Distribution of IGP-lyases (TSA, IGL, BX1, TSA-like, and INS enzymes) among grasses (monocots) basal eudicots and core eudicots. Presence (green dot) or absence (white dot) of a chloroplast localization peptide (chloroplast LP) is reported. *L. galeobdolon* TSA-1: EU747715. *L. galeobdolon* TSA-2: EU747716. *A. squarrosa* TSA: EU747710. Amino acid sequences were aligned with WebPrank and a Maximum Likelihood phylogenetic tree was inferred using iQTree software. The tree was midpoint rooted.

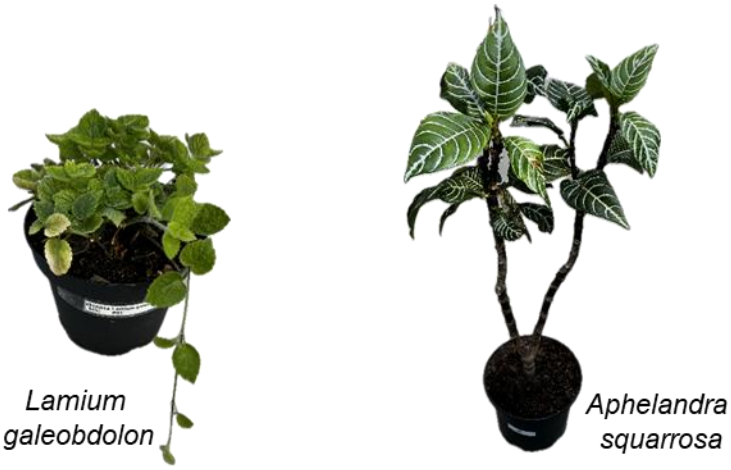

**% nt seq ID**	*LgTSB type II*	*LgTSB*	*LgTSB-like*	*AsTSB-type II*	*AsTSB*	*AsTSB-like*
LgTSB type II	-	38.2	36	76.5	36.4	36.1
LgTSB	38.2	-	56.2	38	79.5	56.3
LgTSB-like	36	56.2	-	34.2	57	69.1
AsTSB-type II	76.5	38	34.2	-	37.1	35.8
AsTSB	36.4	79.5	57	37.1	-	55.6
AsTSB-like	36.1	56.3	69.1	35.8	55.6	-

**% AA seq ID**	*Lg*TSB type II	*Lg*TSB	*Lg*TSB-like	*As*TSB type II	*As*TSB	*As*TSB-like
*Lg*TSB type II	-	21.9	23	78.8	21.2	22.1
*Lg*TSB	21.9	-	56.8	22.4	79.2	54.7
*Lg*TSB-like	23	56.8	-	23.2	54	70.8
*As*TSB type II	78.8	22.4	23.2	-	22.1	22.8
*As*TSB	21.2	79.2	54	22.1	-	53.7
*As*TSB-like	22.1	54.7	70.8	22.8	53.7	-

Supplementary Figure 3: Nucleotide and amino acid sequence identity between TSB-like, TSB, and TSB type II sequences of *Aphelandra squarrosa* (As) and *Lamium galeobdolon* (Lg). Sequences were aligned with MUSCLE 5.1. Plant pictures were taken by the authors.

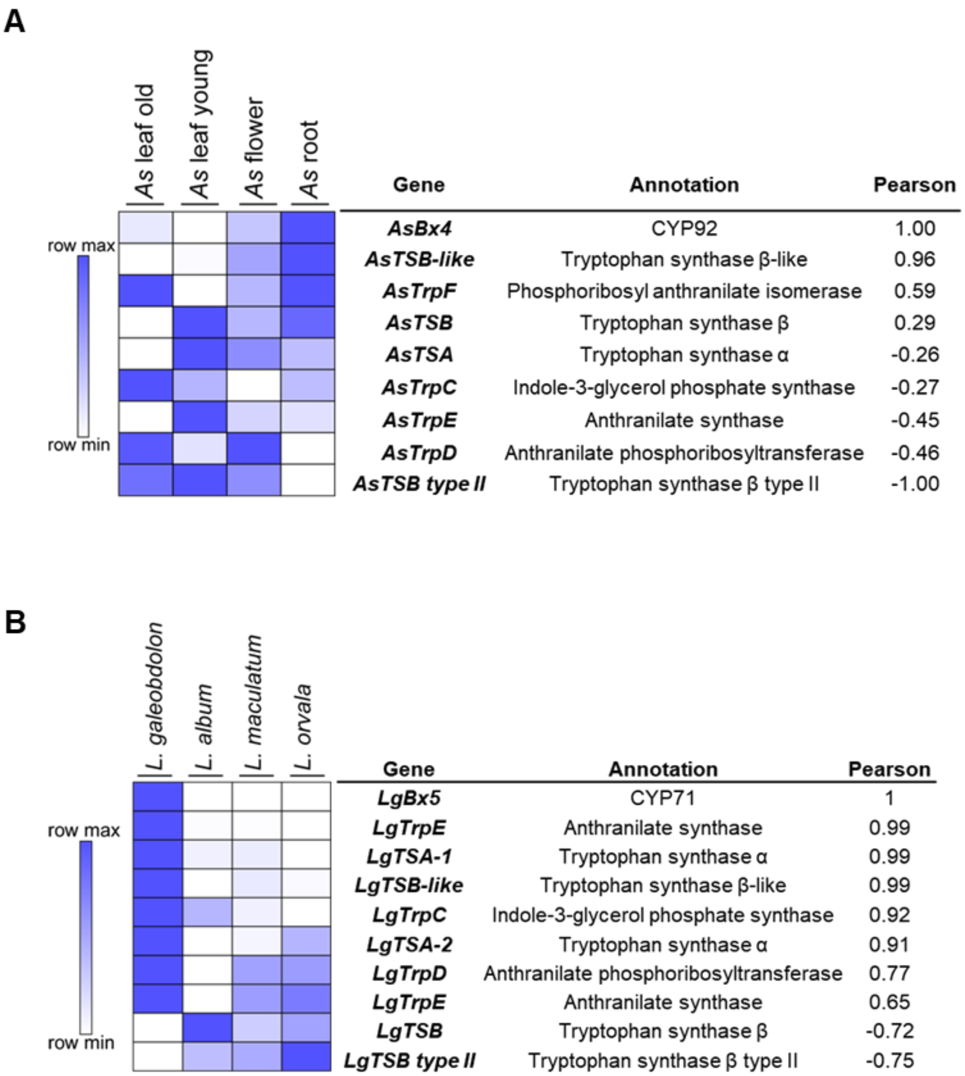

Supplementary Figure 4: TSB-like co-express with *Bx* genes in the BXD-producing species *Aphelandra squarrosa* and *Lamium galeobdolon*. **A**) Heatmap displaying the expression of genes involved in indole and tryptophan biosynthesis in *A. squarrosa* and the corresponding Pearson correlation values with *AsBx4.* **B**) Heatmap displaying the expression of genes involved in indole and tryptophan biosynthesis and the corresponding Pearson correlation values with *LgBx5* in different *Lamium* species*.* Among the displayed species*,* only *L. galeobdolon* is a BXD producer. Note, two *TSA* genes are expressed in *L. galeobdolon*.

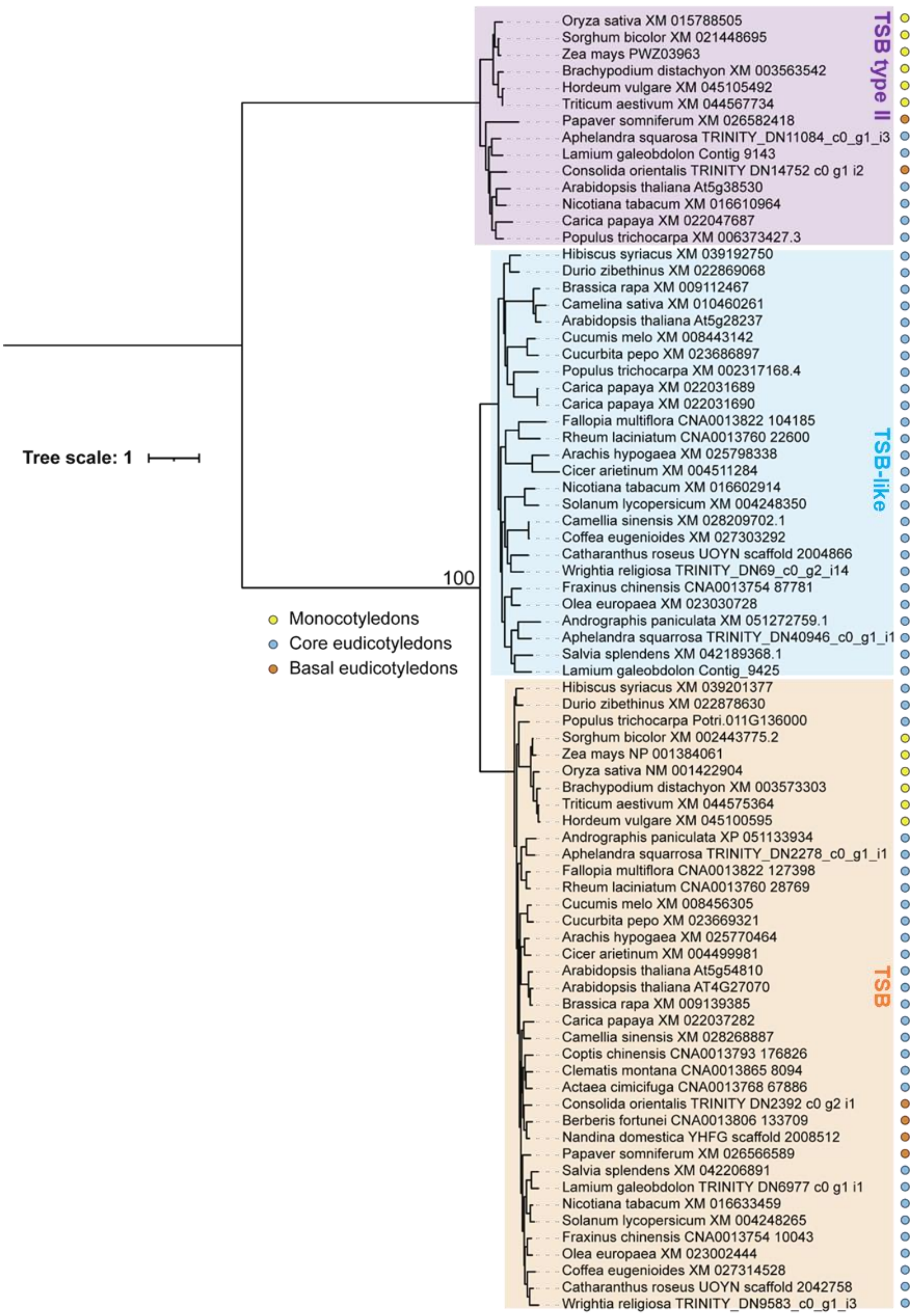

Supplementary Figure 5: Amino acid sequences of TSB, TSB-like, and TSB type II form three separate clades. The occurrence of TSB, TSB-like, and TSB type II sequences in monocots (yellow circle), basal eudicots (orange circle), and core eudicots (blue circle), is reported. TSB and TSB type II are present in monocots, basal eudicots, and core eudicots, while TSB-like occur only in core eudicots. Amino acid sequences were aligned with WebPrank algorithm and a Maximum Likelihood tree was inferred using iQTree.

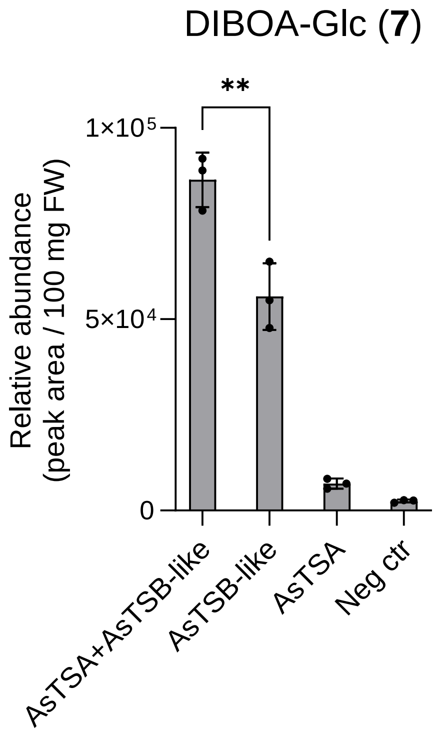

Supplementary Figure 6: Transient expression of *AsTSB-like* and *AsTSA* + *AsTSB-like* in *Nicotiana benthamiana* results in substantial DIBOA-Glc accumulation. *AsTSA* and *AsTSB-like* were transiently expressed in *N. benthamiana* along with maize *Bx2, Bx3, Bx4, Bx5,* and *Bx8* genes. The endogenous *TSA* of *N. benthamiana* is sufficient to obtain high levels of DIBOA-Glc accumulation upon expression of *AsTSB-like*. DIBOA-Glc levels are further increased by co-expression of *AsTSA* and *AsTSB-like*. Bar graphs represent the mean ± SD for three independent biological replicates (n = 3, plants). ** = p<0.01 (two-tailed t-test, p value = 0.0094, t = 4.693, df = 4).

A

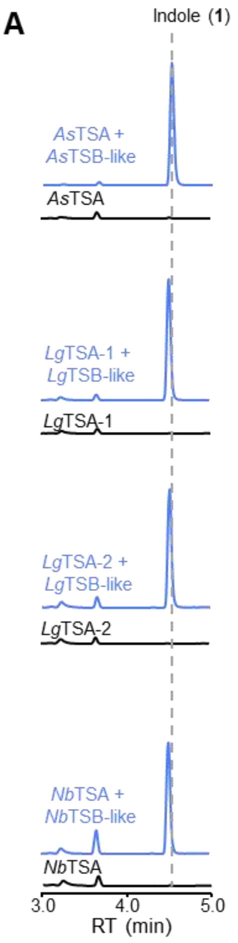

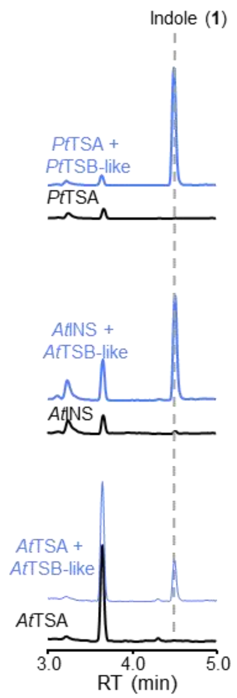

B

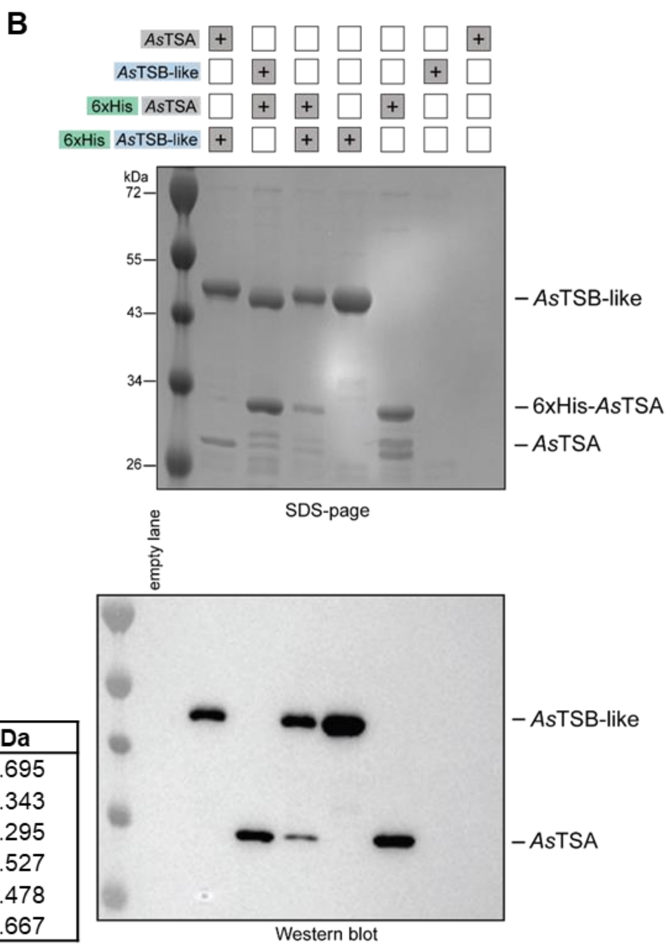

C

Protein	kDa
*As*TSA no tag	29.695
*As*TSA C-His	30.343
*As*TSA N-His	33.295
*As*TSB-like no tag	46.527
*As*TSB-like C-His	47.478
*As*TSB-like N-His	48.667

Supplementary Figure 7: Testing TSA and TSB-like interaction and allosteric activation. **A**) Proteins were expressed in *Escherichia coli* and purified TSA, INS, and TSB-like from *Aphelandra squarrosa*, *Lamium galeobdolon*, *Populus trichocarpa*, *Nicotiana benthamiana,* and *Arabidopsis thaliana* were assayed with IGP. Indole produced by TSA/INS alone (black) and indole produced by TSA/INS + TSB-like (blue) was measured using liquid chromatography-tandem mass spectrometry. **B**) *N*-terminal His tagging of *As*TSA and *As*TSB-like allows TSA-TSB-like complex formation. *E. coli* cultures expressing *N*-terminal His-tagged or untagged TSA and TSB-like were mixed and His-tagged proteins were retrieved trough affinity purification. Untagged TSA or TSB-like co-purified with the corresponding tagged partner (SDS-page) although only one protein was His-tagged (Western-blot). **C**) Table with the size of *As*TSA and *As*TSB-like with N and C-terminal 6xHis tag or untagged.

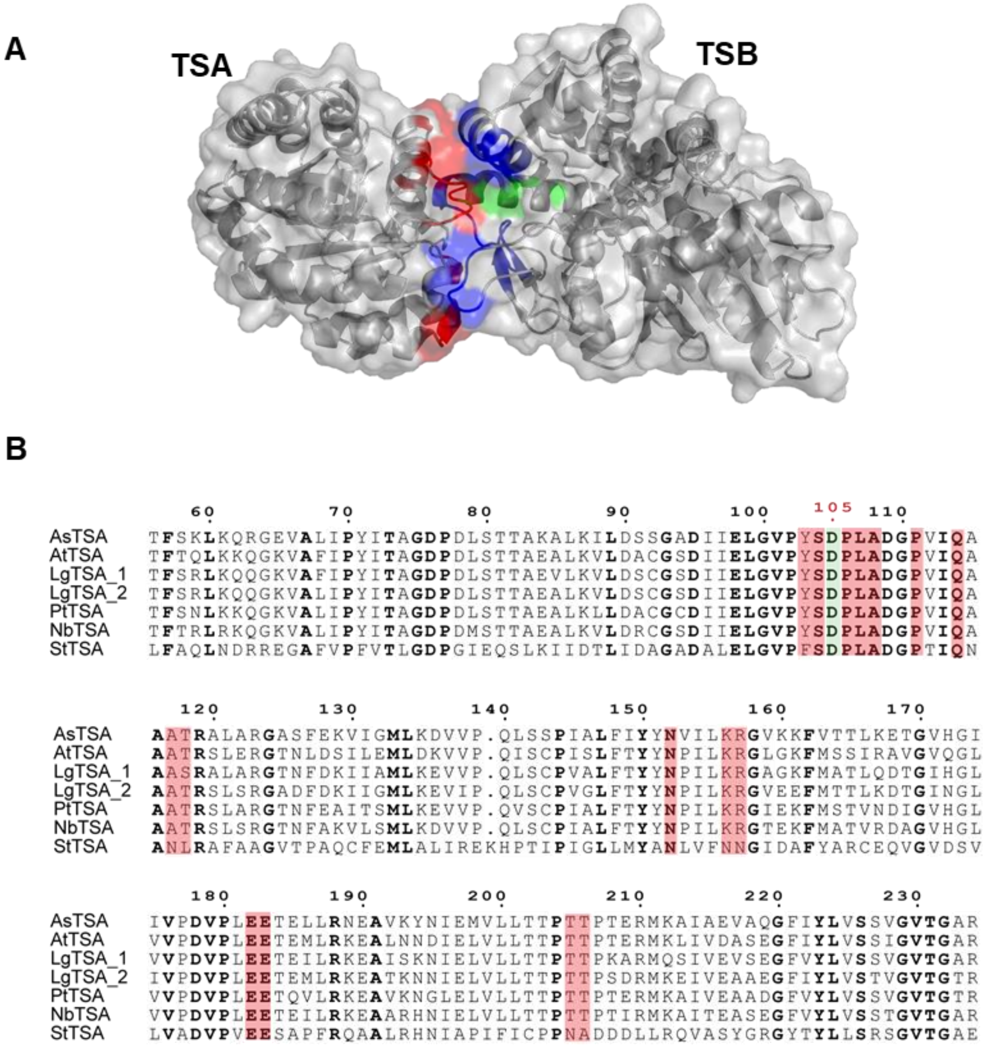

Supplementary Figure 8: Residues forming the TSA-TSB interface. **A)** Crystal structure of the TSA-TSB complex from *Salmonella typhimurium* (PDB: 1BKS). Residues located at the interface between TSA and TSB are highlighted in red (TSA) and in blue (TSB). Residues highlighted in green in both proteins indicate residues experimentally shown to interact between TSA and TSB (1). **B)** Sequence alignment of *Aphelandra squarrosa* TSA (*As*TSA), *Arabidopsis thaliana* TSA (*At*TSA), *Lamium galeobdolon* TSA_1 (*Lg*TSA_1), *L. galeobdolon* TSA_2 (*Lg*TSA_2), *Populus trichocarpa* TSA (*Pt*TSA), *Nicotiana benthamiana* TSA (*Nb*TSA), and *Salmonella typhimurium* TSA (*St*TSA). The figure displays exclusively the parts of the alignment containing TSA residues (highlighted in red) present at the TSA-TSB interface. Residues experimentally shown to interact between TSA and TSB are highlighted in green (1).

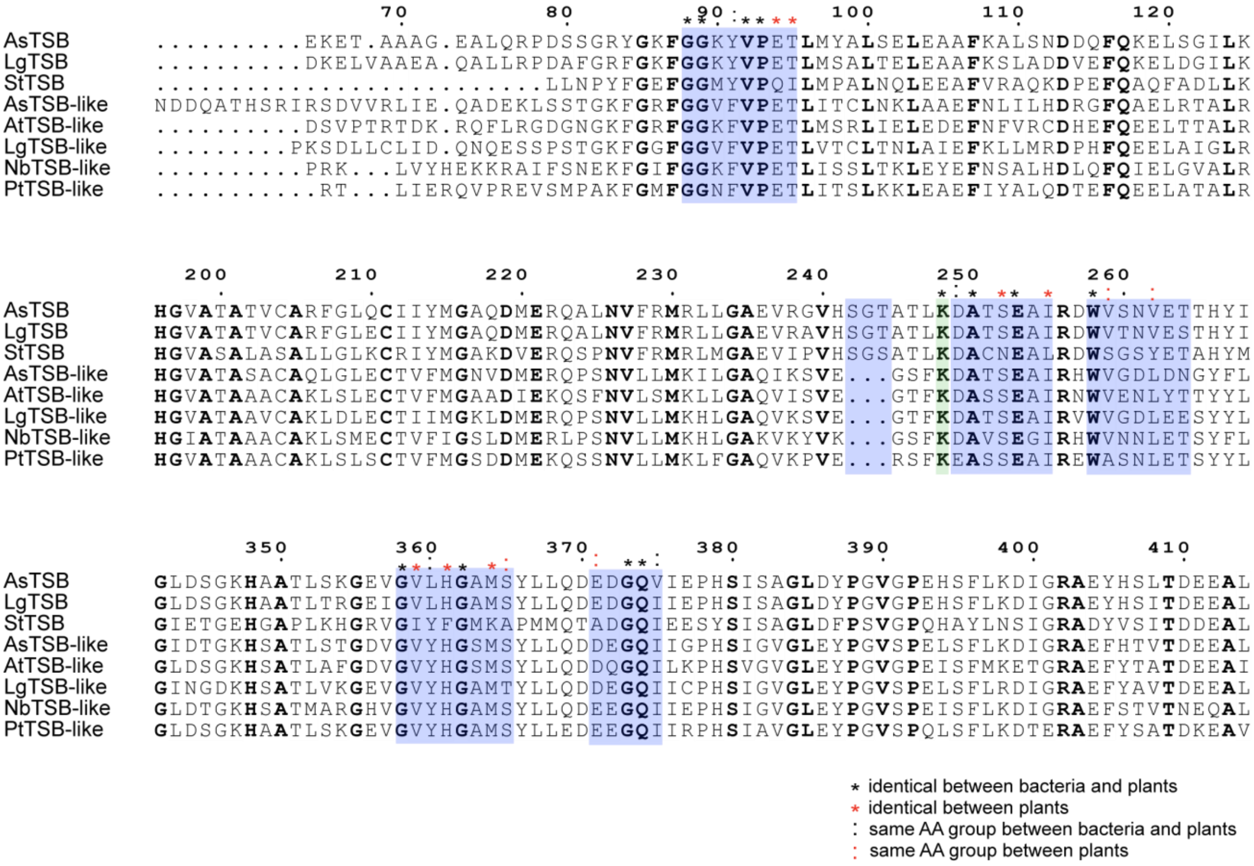

Supplementary Figure 9: TSB residues forming the interface with TSA**.** Alignment of TSB and TSB-like sequences from *Aphelandra squarrosa* (*As*TSB and *As*TSB-like), *Arabidopsis thaliana* (*At*TSB and *At*TSB-like), *Lamium galeobdolon* (*Lg*TSB and *Lg*TSB-like), *Populus trichocarpa* (*Pt*TSB and *Pt*TSB-like), *Nicotiana benthamiana* (*Nb*TSB and *Nb*TSB-like), and *Salmonella typhimurium* (*St*TSB). The figure displays exclusively the parts of the alignment containing residues present at the TSA-TSB interface. Residues forming the interface with TSA are highlighted in blue. Residues highlighted in green indicate residues experimentally shown to interact between TSA and TSB (1).

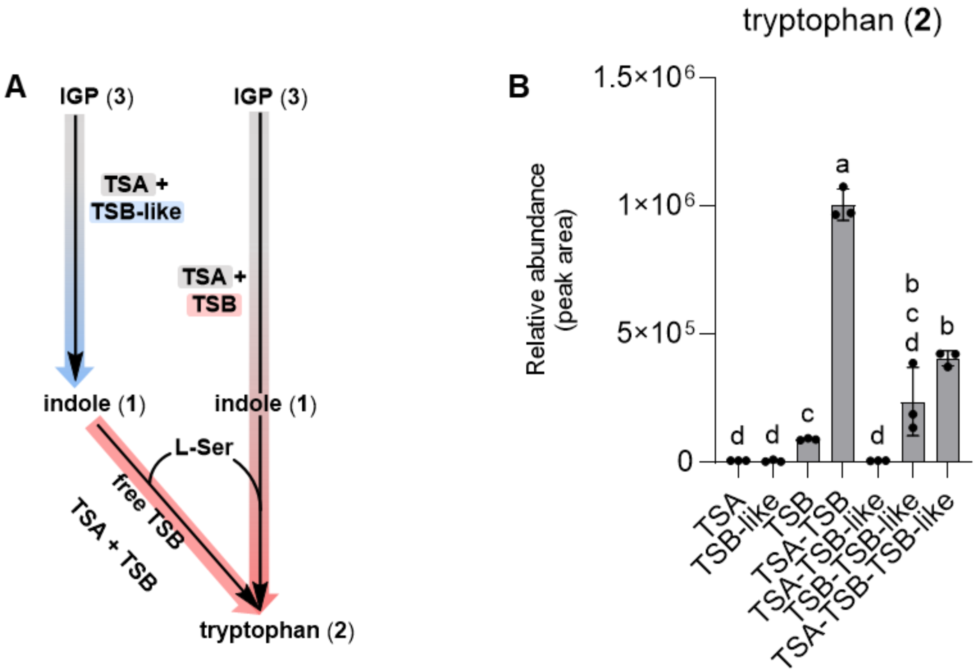

Supplementary Figure 10: Indole can be used as a substrate both from TSA-TSB complex as well as from TSB alone. **A**) Scheme depicting the cross talk between the product of TSA-TSB-like reaction (indole), which act as a substrate for tryptophan biosynthesis by TSA-TSB or in minor amount for TSB alone. The cross-talk between the product and substrate of the two reactions explains the tryptophan increase concomitant with indole increase in Fig 2D iii and iv. **B**) Both, the TSA-TSB complex as well as TSB alone produce tryptophan from free indole and L-Ser. Co-incubation of TSB and TSB-like in the presence of indole and L-serine did not result in significantly altered tryptophan formation compared to TSB alone. Proteins were expressed in *E. coli*, purified, and assayed on indole and L-Ser. Reaction products were analyzed using liquid chromatography-tandem mass spectrometry. Bar graphs represent the mean ± SD for three technical replicates (n = 3, assays). Columns labeled with different letters represent statistically significant differences ($p < 0.05$, Brown-Forsythe and Welch ANOVA with Dunnett test for multiple comparison). Detailed statistical values for each comparison are reported in Supplementary Data 1.

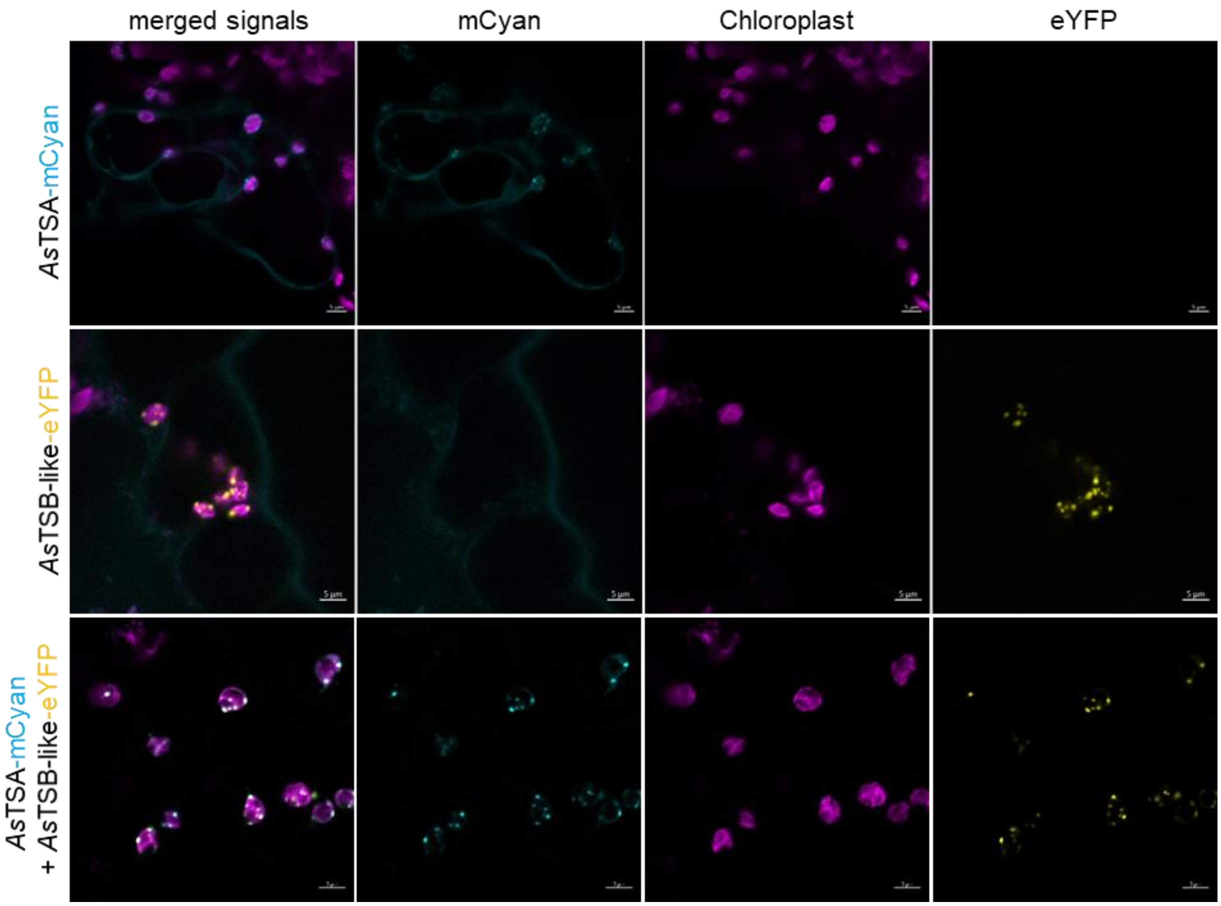

Supplementary Figure 11: Subcellular localization of *As*TSA and *As*TSB-like. *As*TSA fused to a *C*-terminal mCyan and *As*TSB-like fused to a C-terminal eYFP were transiently expressed in *Nicotiana benthamiana* under control of the *Solanum lycopersicum* Ubq10 promoter and terminator. Chloroplast localization was assessed using chloroplast autofluorescence. *N. benthamiana* was transiently transformed with the single *AsTSA* or *AsTSB-like* construct or co-infiltrated with both.

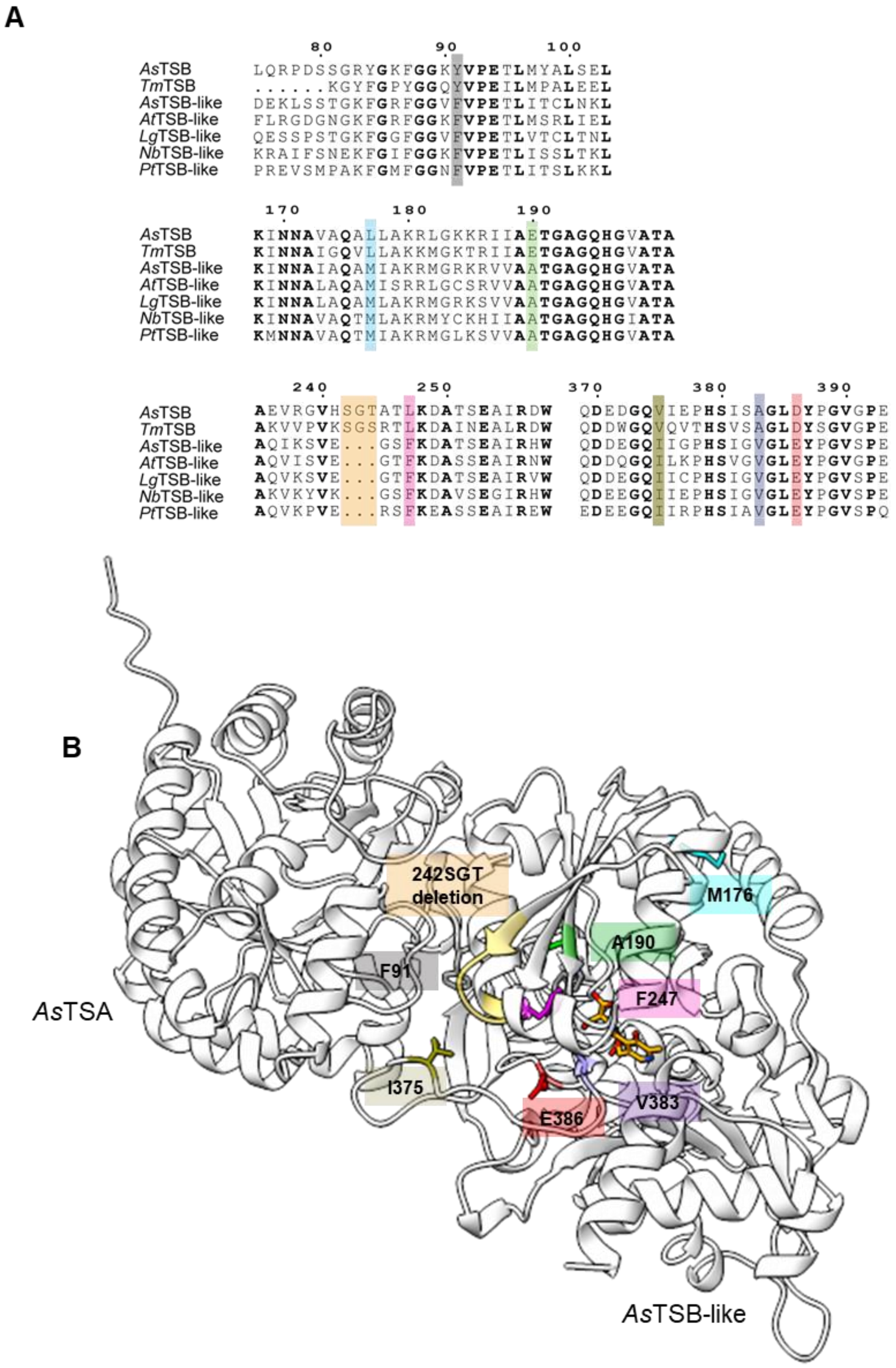

Supplementary Figure 12: Highly conserved residues that differ between TSB and TSB-like. **A**) Muscle alignment displaying *Aphelandra squarrosa* (*As*), *Arabidopsis thaliana* (*At*), *Lamium galeobdolon* (*Lg*), *Populus trichocarpa* (*Pt*), *Thermotoga maritima* (*Tm*) TSB-like or TSB. **B**) Residues highlighted in (A) are shown in a model of *As*TSA-*As*TSB-like complex. The complex was modelled as a multimer using Alphafold MMSeq.

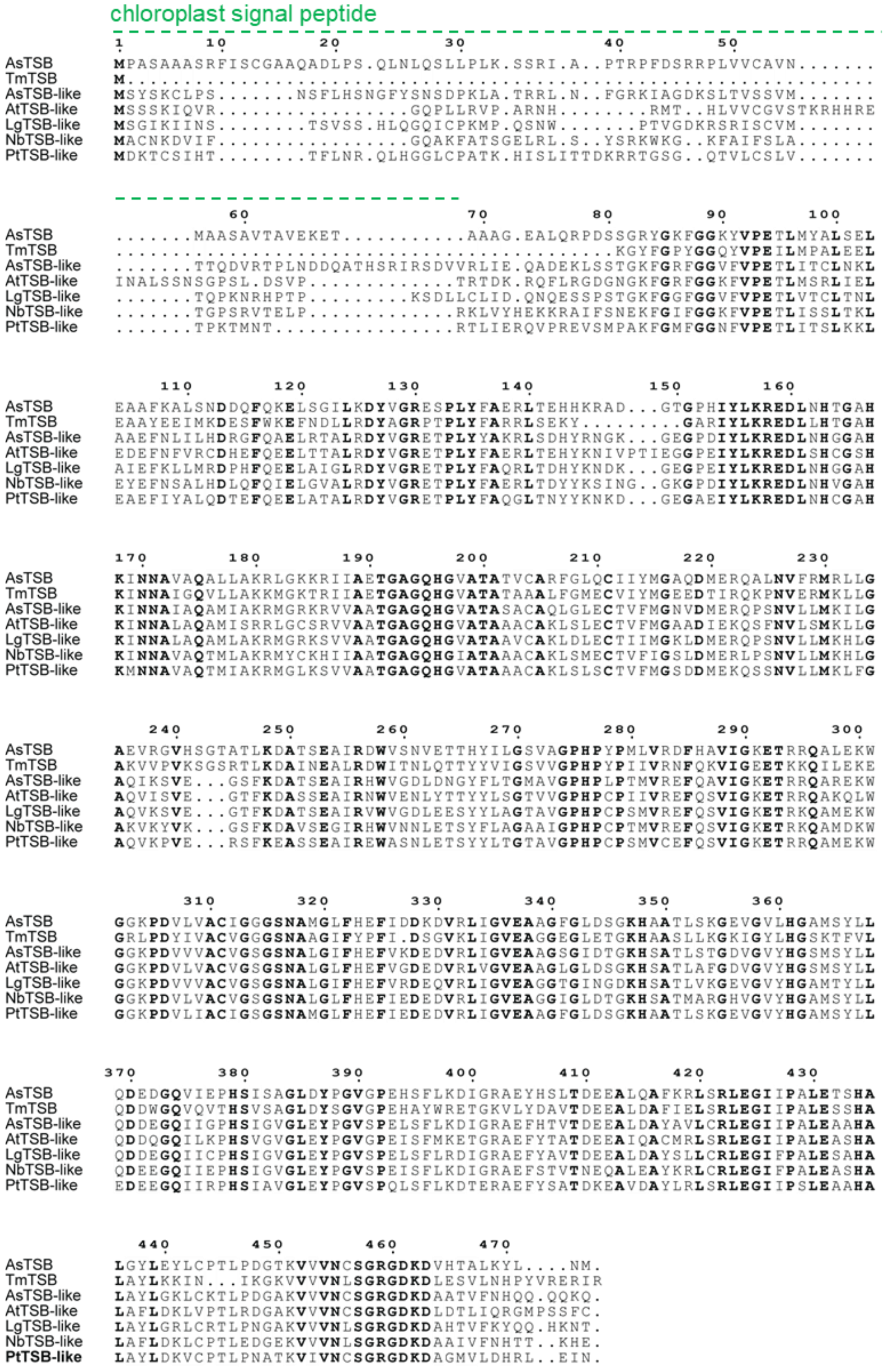

Supplementary Figure 13: TSB-like reference alignment**.** Sequence alignment of TSB and TSB-like sequences from: *Aphelandra squarrosa* (*As*TSB-like and *As*TSB), *Arabidopsis thaliana* (*At*TSB-like), *Lamium galeobdolon* (*Lg*TSB and *Lg*TSB-like), *Populus trichocarpa* (*Pt*TSB- like), *Nicotiana benthamiana* (*Nb*TSB-like), and *Thermotoga maritima* TSB (*Tm*TSB). Sequences were aligned with MUSCLE. For plant sequences, the extension of the chloroplast localization peptide is marked in green.

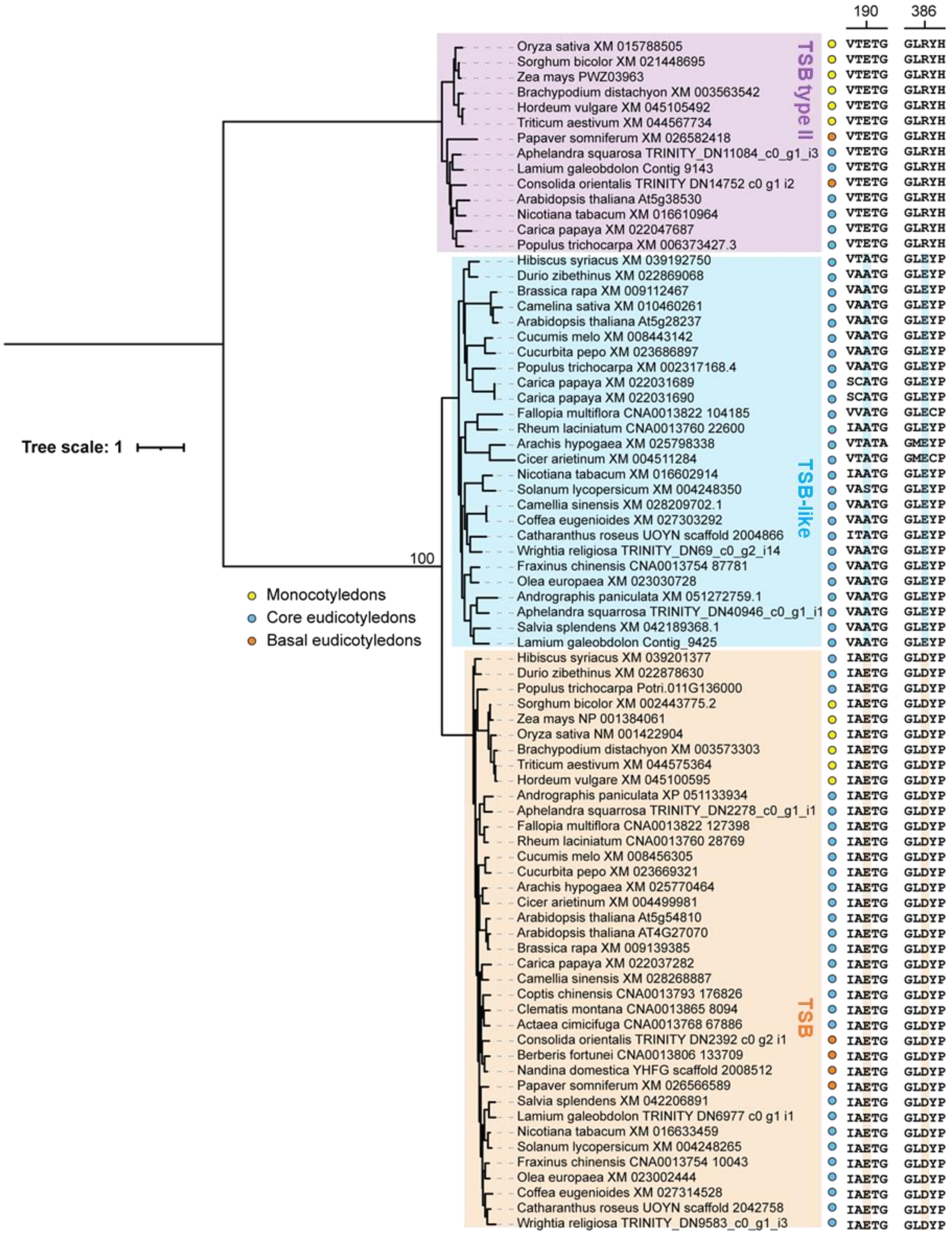

Supplementary Figure 14: Mutation of two conserved residues in TSB-like and TSB leads to activity changes. Phylogenetic tree displaying conservation of residues 190 and 386 among different classes of TSBs. Residues are highlighted with colors according to the TSB clade they belong to. *Solanum lycopersicum* displays a serine instead of the otherwise conserved alanine 190. Amino acid sequences were aligned with WebPrank algorithm and a Maximum Likelihood tree was inferred using iQTree.

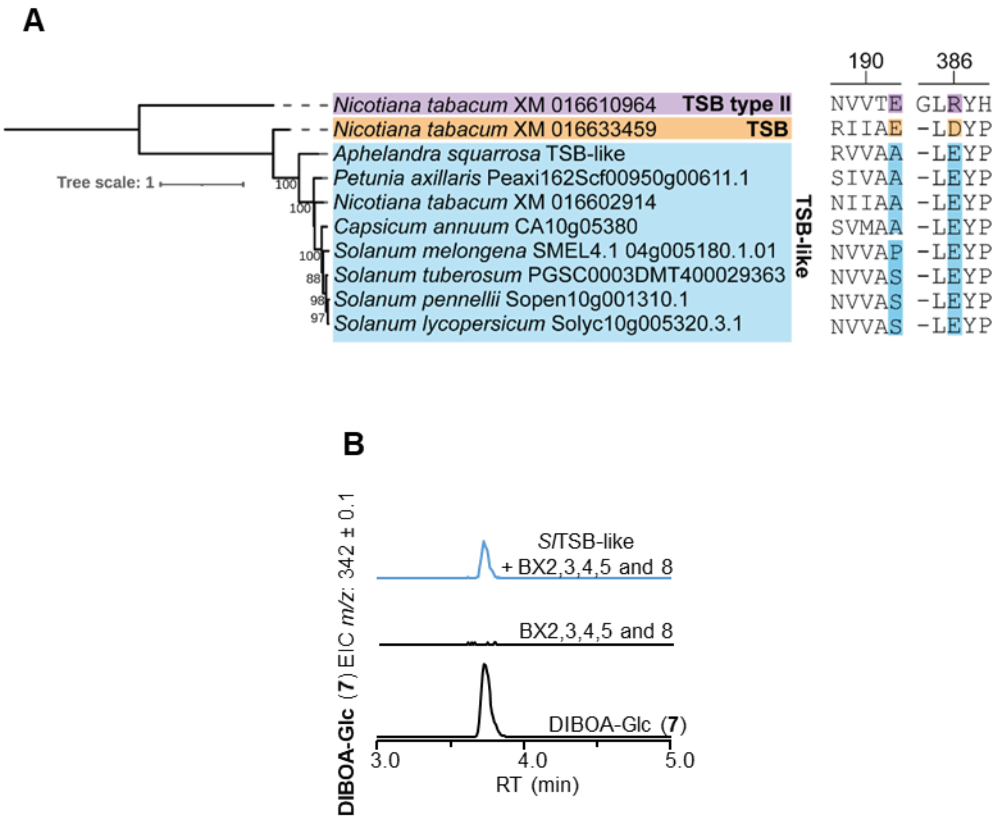

Supplementary Figure 15: A190 is not strictly required for TSB-like activity. **A**) Phylogenetic tree displaying the conservation of residue 190 and 386 among the Solanaceae. *As*TSB-like is used as a reference sequence to mark the TSB-like clade. While most TSB-like proteins have an alanine at position 190, members of the *Solanum* genus have either a proline or serine residue at this position. E386 is conserved in all TSB-like. The tree was constructed using amino acid sequences, alignment was performed with WebPrank, and a Maximum Likelihood tree was inferred using iQtree. **B**) *S. lycopersicum TSB-like* was expressed in *N. benthamiana* with *Z. mays Bx2, Bx3, Bx4, Bx5* and *Bx8*. Leaf methanolic extracts were analyzed using liquid chromatography-time-of-flight mass spectrometry and DIBOA-Glc accumulation was confirmed with an authentic standard.

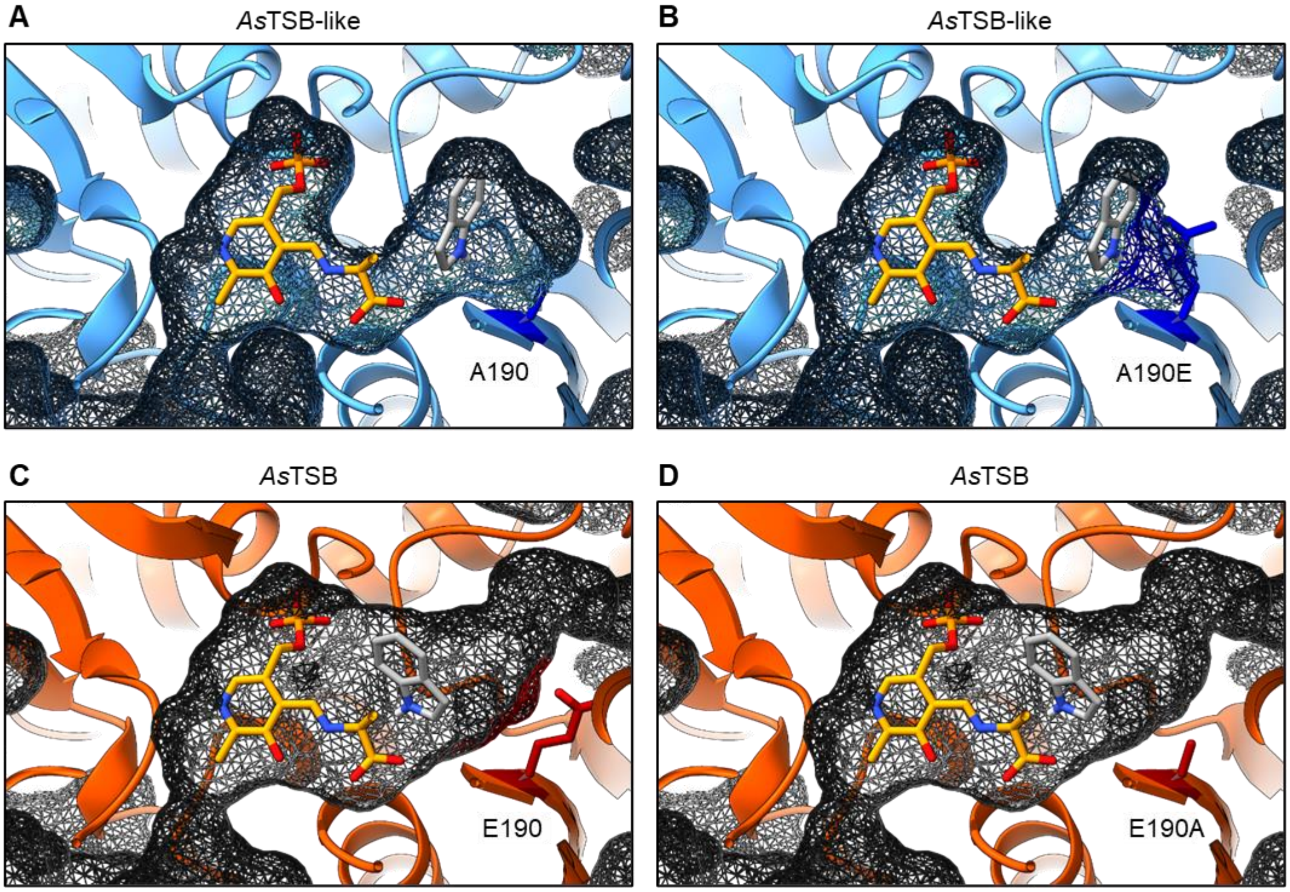

Supplementary Figure 16: Active site of *A. squarrosa* TSB-like (AsTSB-like) and *A. squarrosa* TSB (AsTSB) and effect of site-directed mutagenesis of residue 190 on the active site architecture. Protein structures were modelled on the crystal structure of *Salmonella typhimurium* TSB (PDB: 7JMQ) in open conformation with pyridoxal phosphate (yellow). Indole (gray) was docked in silico. A) Active site architecture of *As*TSB-like. B) Active site architecture of *As*TSB-like mutant A190E. C) Active site architecture of *As*TSB. D) Active site architecture of *As*TSB mutant E190A.

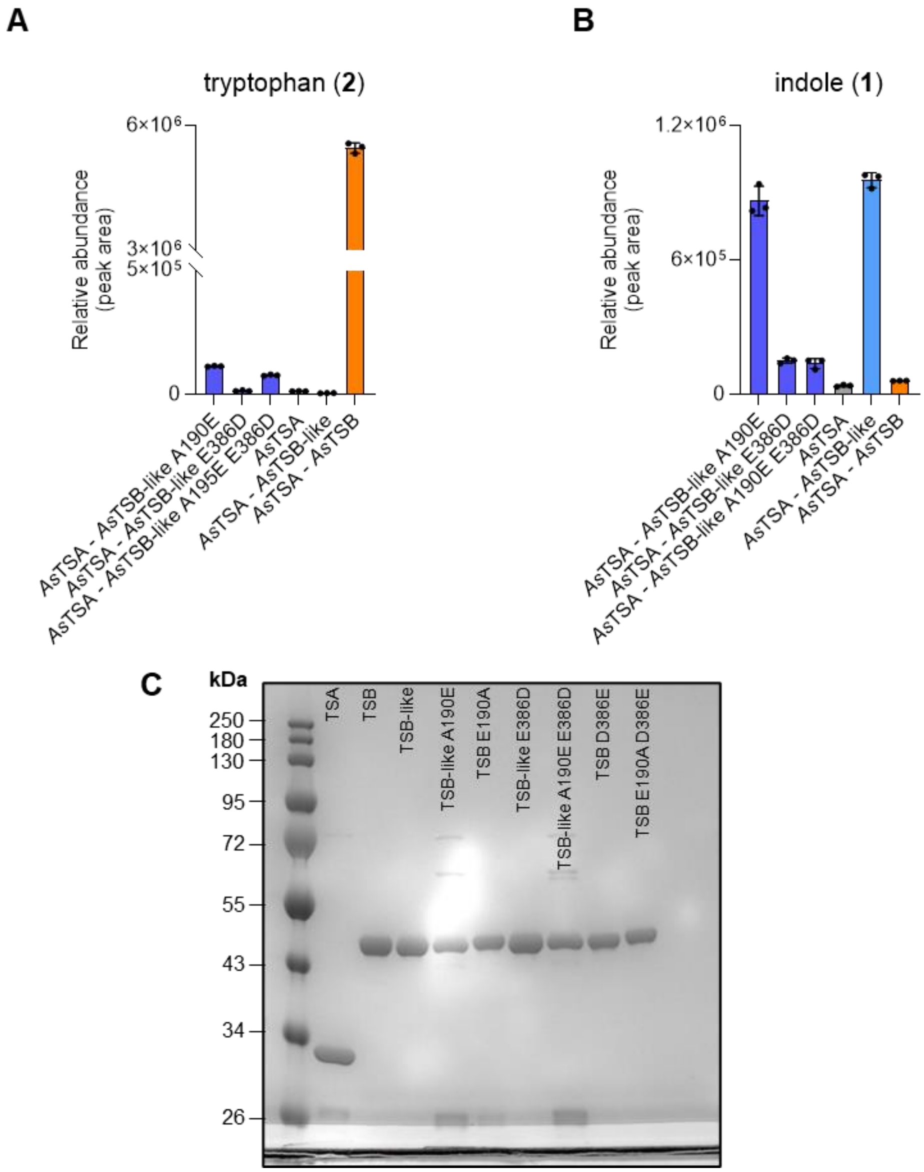

Supplementary Figure 17: Site directed mutagenesis of two conserved residues in TSB-like and TSB leads to activity changes in vitro. Tryptophan (**A**) and indole (**B**) biosynthetic activity of *As*TSB-like mutants. Proteins were expressed in *E. coli*, purified, and assayed with IGP (3) and L-serine. Reaction products were analyzed using liquid chromatography/tandem mass spectrometry. Bar graphs represent the mean ± SD for three technical replicates (n = 3, assays). **C**) SDS-Page gel showing purified *As*TSA, *As*TSB, *As*TSB-like, *As*TSB-like A190E, *As*TSB E190A, *As*TSB-like E386D, *As*TSB-like A190E E386D, *As*TSB D386E and, *As*TSB E190A D386E.

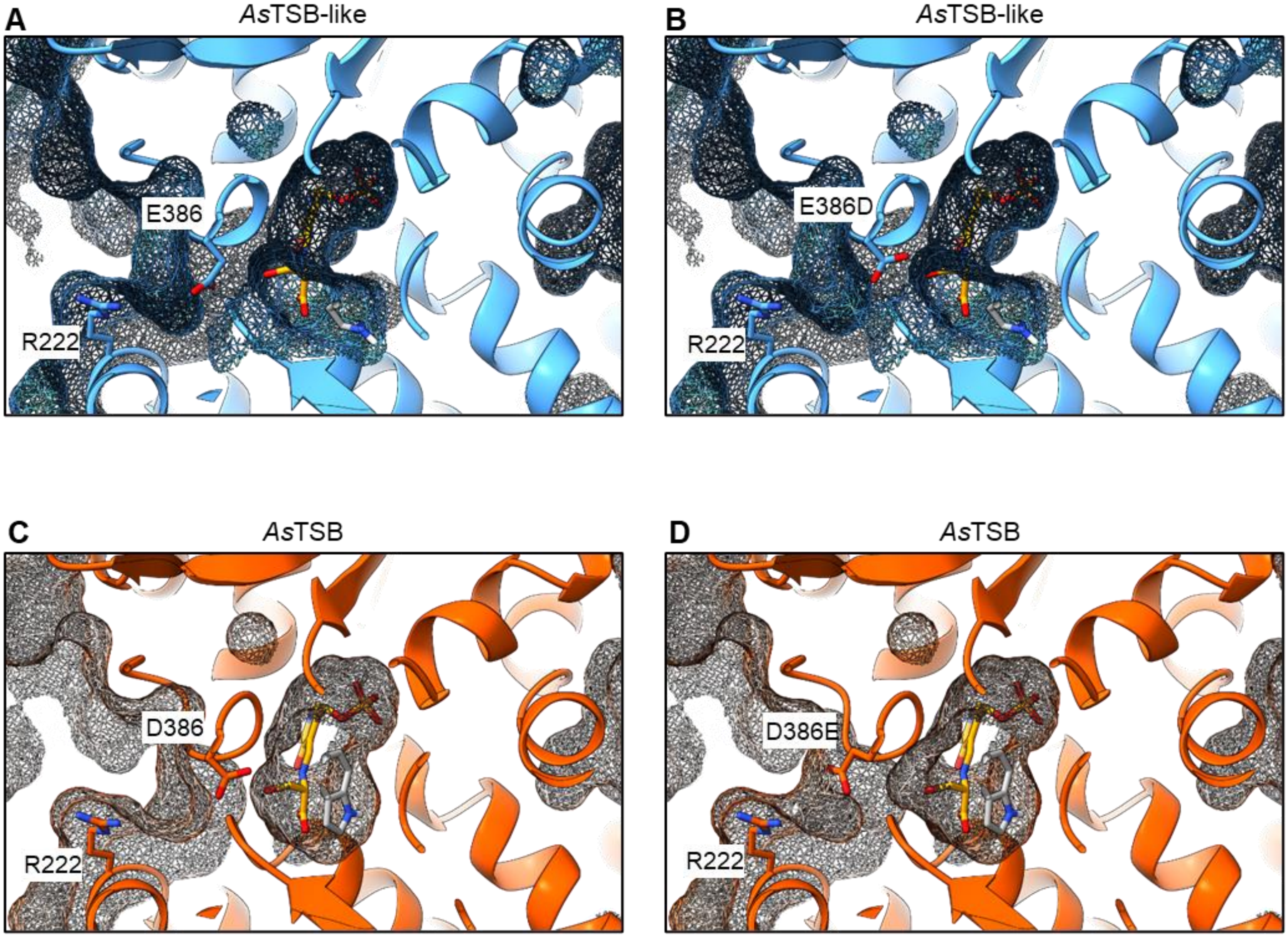

Supplementary Figure 18: Active site of *A. squarrosa* TSB-like (AsTSB-like) and *A. squarrosa* TSB (AsTSB) and effect of site-directed mutagenesis at position 386 on the active site architecture. Protein structures were modelled on the crystal structure of *Salmonella typhimurium* TSB (PDB: 7JMQ) in open conformation with pyridoxal phosphate (yellow). Indole (gray) was docked in silico. A) Active site architecture of *As*TSB-like. B) Active site architecture of *As*TSB-like mutant E386D. C) Active site architecture of *As*TSB. D) Active site architecture of *As*TSB mutant D386E.

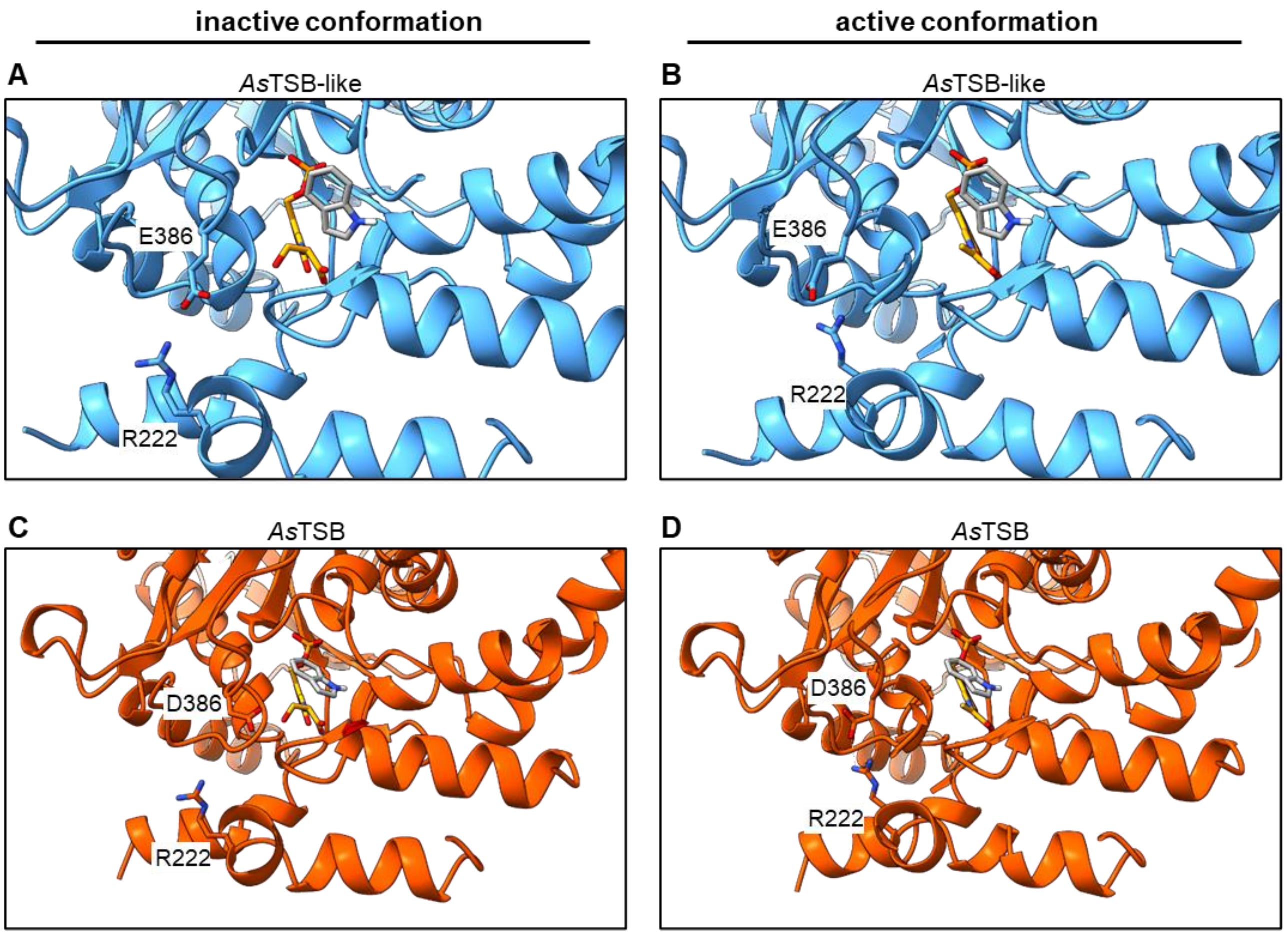

Supplementary Figure 19: Orientation of residue 386 and 222 in the inactive and active conformation of *A. squarrosa* TSB-like and TSB. Protein structures were modelled on the crystal structure of *Salmonella typhimurium* TSB (PDB: 7JMQ) in inactive conformation and of *Salmonella typhimurium* TSB (PDB: 4HPX) in active conformation with pyridoxal phosphate (yellow). Indole (gray) was docked in silico. A) Predicted orientation of residue E386 and R222 in *As*TSB-like modelled in inactive conformation. B) Predicted orientation of residue E386 and R222 in *As*TSB-like modelled in active conformation. C) Predicted orientation of residue D386 and R222 in *As*TSB modelled in inactive conformation. D) Predicted orientation of residue D386 and R222 in *As*TSB modelled in active conformation. In the structure of TSB enzymes in active conformation, the side chain of residue D386 is known to undergo a significant rotation, pointing away from the active site and forming a salt bridge with residue R222. This interaction is known to stabilize the active conformation. Notably, homology modelling of *As*TSB-like on *S. typhimurium* crystal structure always predicted this residue to be oriented away from the active site, and towards R222, even in the inactive conformation.

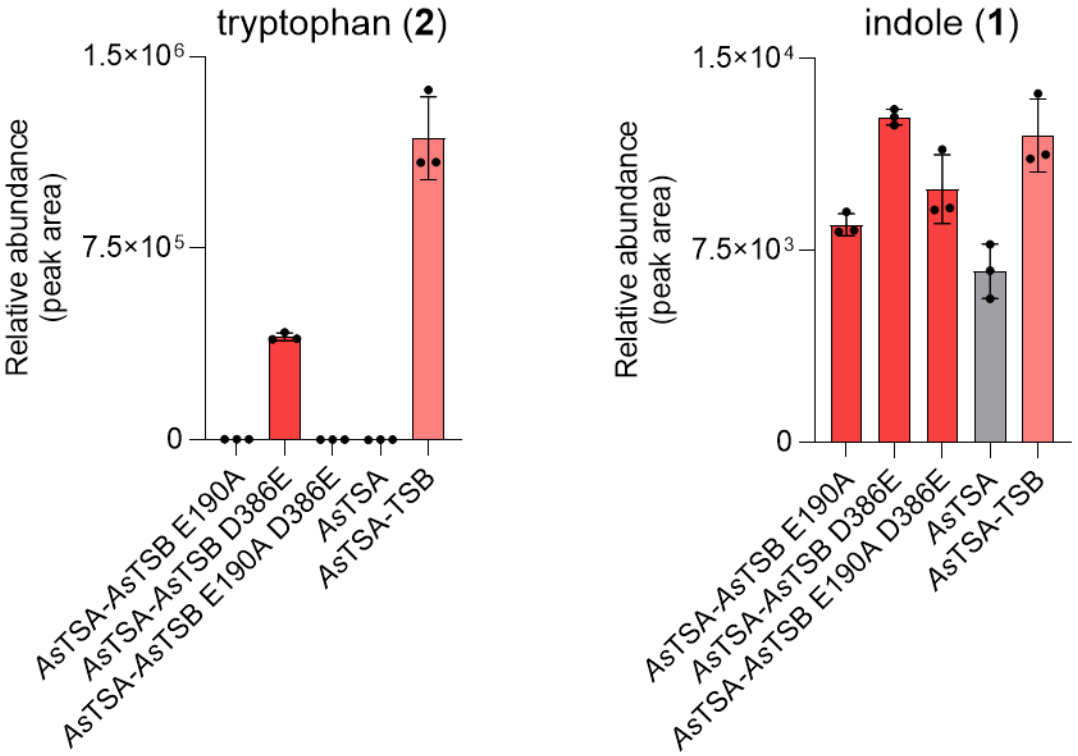

Supplementary Figure 20: Site-directed mutagenesis of E190A and D386E is not sufficient to convert *As*TSB into *As*TSB-like. *As*TSB E190A showed loss of tryptophan biosynthetic activity, while *As*TSB D386E showed reduced but still significant tryptophan biosynthesis without a significant gain in indole formation. The double mutant *As*TSB E190A D386E did not show tryptophan biosynthetic activity, but neither gain of indole biosynthetic activity. Proteins were expressed in *Escherichia coli*, purified, and assayed with IGP and L-serine. Reaction products were analyzed using liquid chromatography-tandem mass spectrometry. Bar graphs represent the mean ± SD for three technical replicates (n = 3, assays).

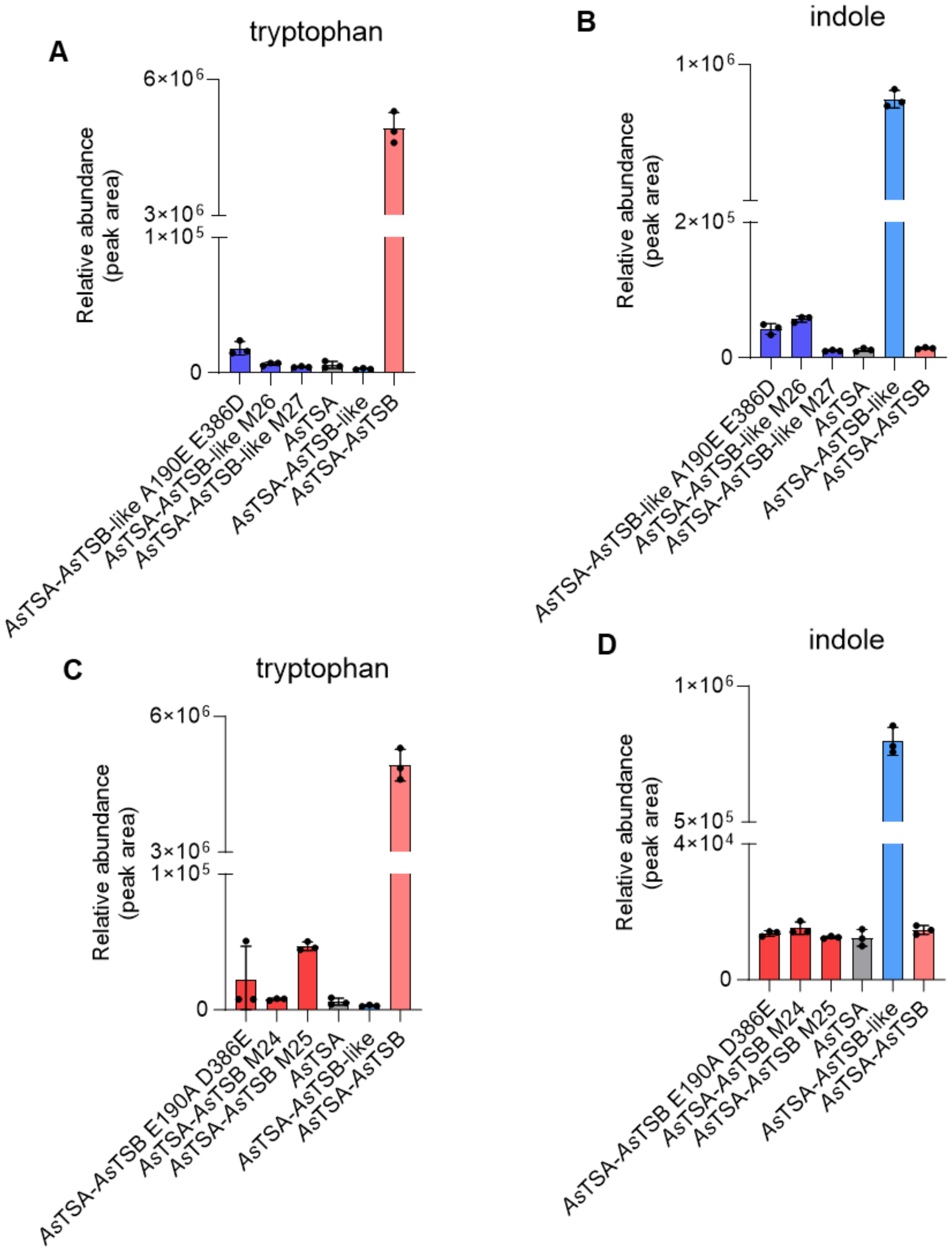

Supplementary Figure 21: Site directed mutagenesis of highly conserved residues that differ between TSB and TSB-like clades did not substantially change the activity of the resulting proteins. **A** and **B**) Extended mutagenesis of residues identified in Supplementary Fig.12 on *As*TSB-like background did not improve tryptophan biosynthetic activity. TSB-like M26 consisted of *As*TSB-like containing A190E, F247L, V383A, E386D site directed mutations. TSB-like M27 consisted of *As*TSB-like containing F91Y, M177L, A190E, 242SGT insertion, F247L, I375V, V383A, E386D site directed mutations. **C** and **D**) Extended mutagenesis on *As*TSB background did not improve indole biosynthetic activity. M24 consisted of *As*TSB containing E190A, L247F, A383V, D386E site directed mutations. M25 consisted of *As*TSB containing Y91F, L177M, E190A, 242SGT deletion, L247F, V375I, A383V, D386E site directed mutations. Proteins

were expressed in *E. coli*, purified, and tested in combination with *As*TSA on IGP and L-serine. Reaction products were analyzed using liquid chromatography-tandem mass spectrometry. Bar graphs represent the mean ± SD for three technical replicates (n = 3, assays).

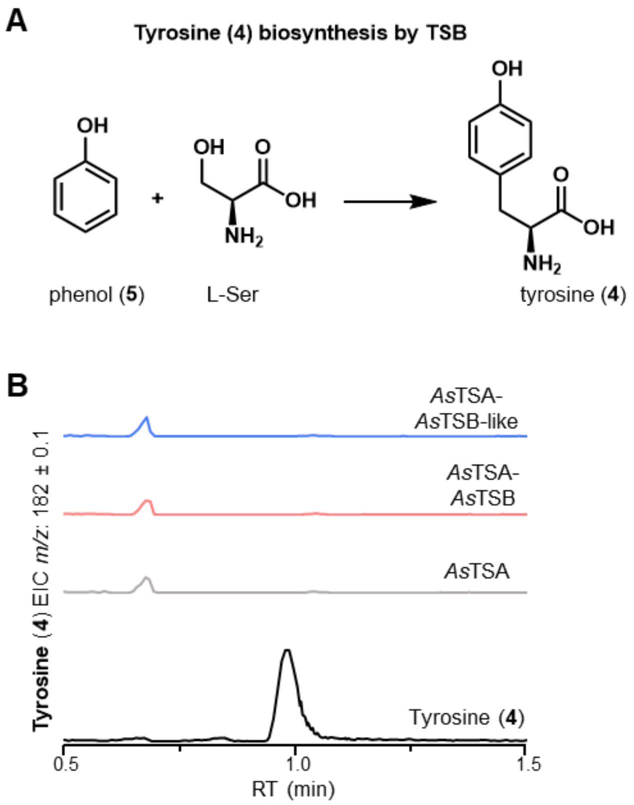

Supplementary Figure 22: *As*TSB-like shows no tyrosine biosynthetic activity. **A**) Tyrosine formation catalyzed by TSB as reported by (2). **B**) *As*TSB-like and *As*TSB show no tyrosine biosynthetic activity. Proteins were expressed in *Escherichia coli*, purified, and assayed with phenol and L-serine. Reactions were quenched with MeOH:1MHCl and reaction products were analyzed using liquid chromatography-time-of-flight mass spectrometry. Extracted ion chromatograms (EIC) are shown.

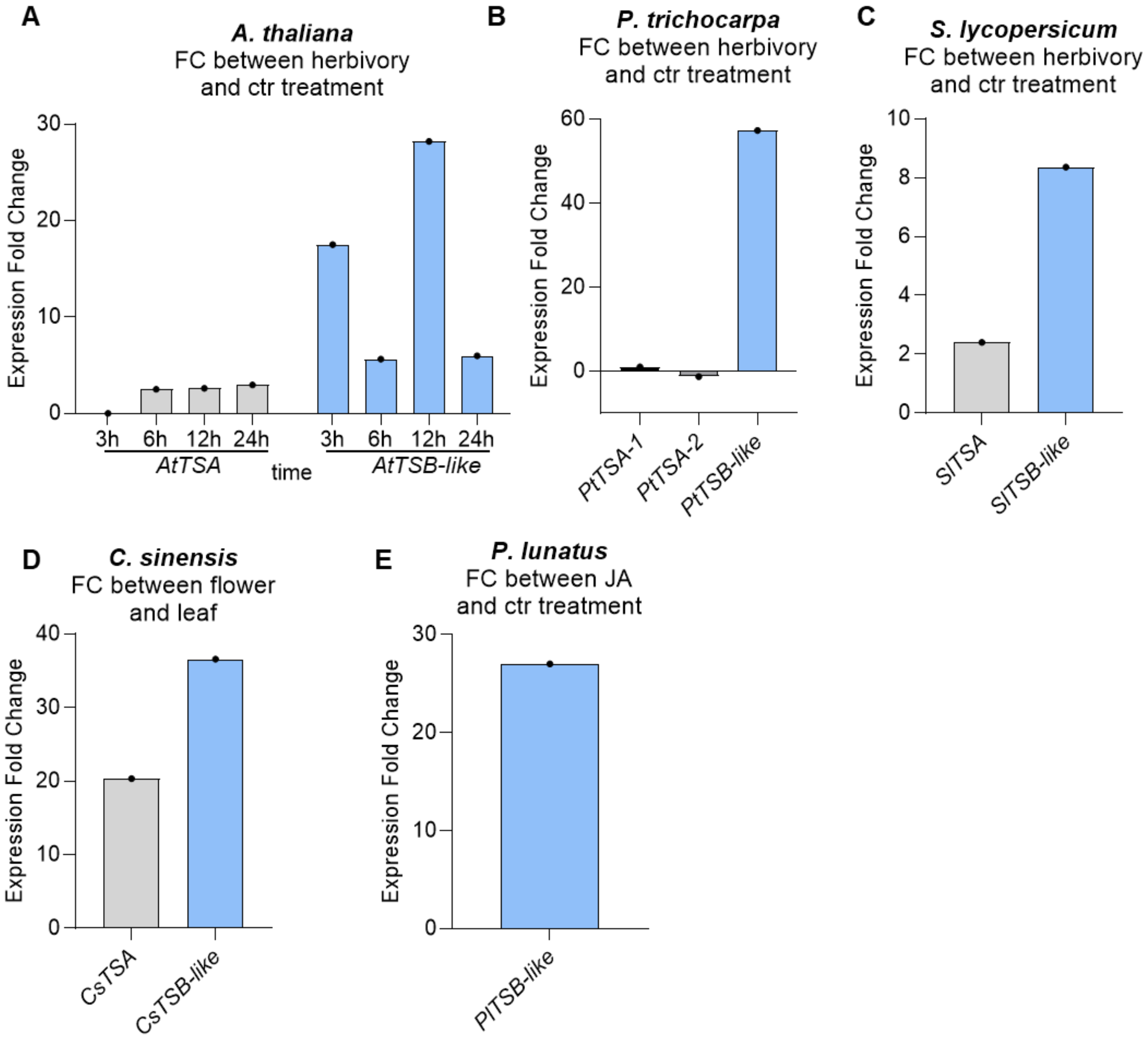

Supplementary Figure 23: Expression differences of TSA and TSB-like genes between conditions in which indole emission is induced and the control treatment in different plant species. Expression differences are showed as Fold Change (FC). **A**) FC of *Arabidopsis thaliana TSA* and *TSB-like* over the course of 24 h exposure to *Pieris rapae* caterpillars as reported by (3). **B**) FC of *Populus trichocarpa TSA* and *TSB-like* after 24 h of *Lymantria dispar* caterpillar feeding as reported by (4). **C**) FC of *Solanum lycopersicum TSA* and *TSB-like* after 40 days of *Tuta absoluta* exposure as reported by (5). **D**) FC of *Citrus sinensis* (sweet orange) *TSA* and *TSB-like* between the indole-producing flowers and the indole non-producing leaves (www.orangeExpDB.com). **E**) FC of *Phaesous lunatus* TSB-like between jasmonic acid (JA) and control treatment. No *TSA* was reported as differentially expressed in *P. lunatus* plants treated with JA as reported by (6).

A

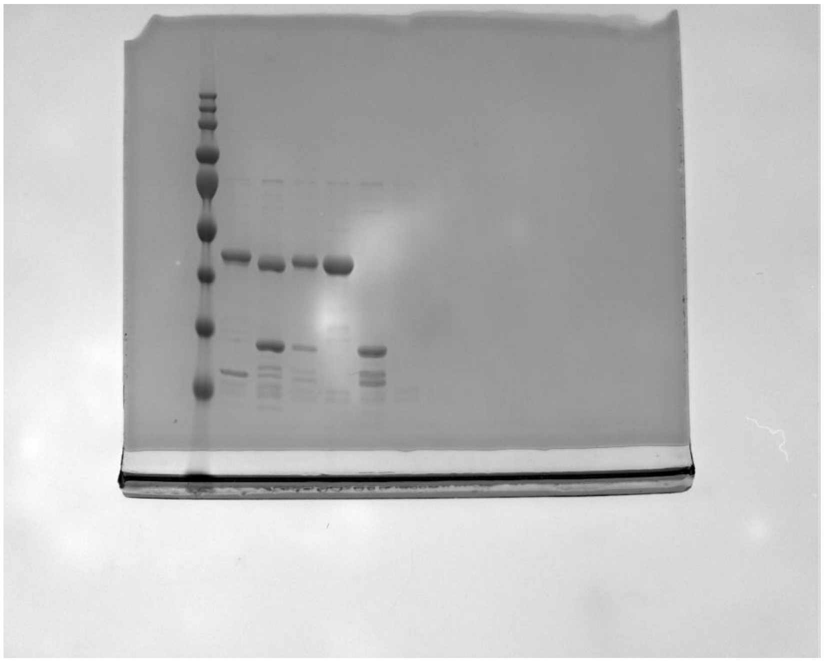

B

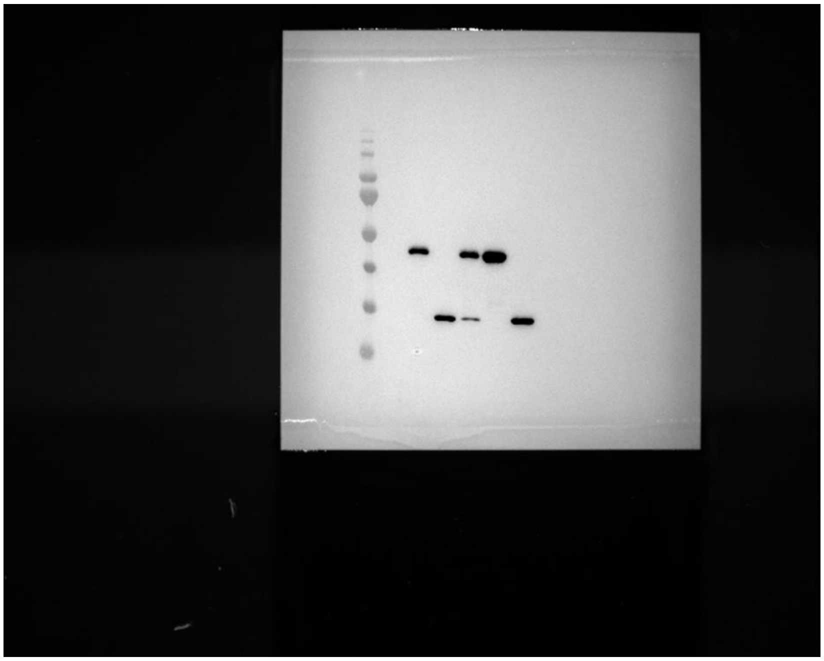

Supplementary Figure 24: Source Data File for Supplementary Figure 7B. **A**) Uncropped image of SDS-page. **B**) Uncropped image of Western blot. Illuminated image of the membrane was superimposed on luminescence image for protein marker alignment.

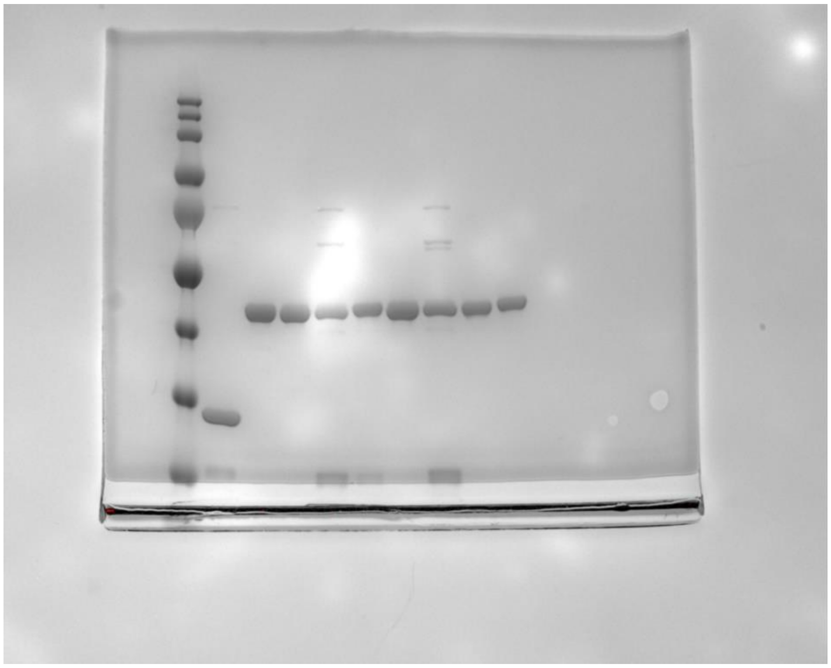

Supplementary Figure 25: Source Data File for Supplementary Figure 17C. Uncropped image of SDS-page.

Supplementary Table 1: Accession and sequences of the genes characterized in this study.

Sequence name	Accession	Sequence
Aphelandra squarrosa TSB-like	PQ246688	ATGTCCTATAGTAAATGTCTTCCCTCCAATTCCTTCCTCCATTC<br>CAACGGCTTCTACTCTAATTCTGACCCCAAGTTGGCAACCCGG<br>CGCCTCAACTTCGGCCGGAAAATTGCAGGAGATAAATCCTTGA<br>CAGTGAGTTCAGTGATGACGACTCAGGACGTGCGTACCCCGT<br>TAAATGATGATCAGGCTACTCATTCGAGGATAAGGTCCGACGT<br>GGTCCGGCTGATCGAACAGGCCGACGAGAAGCTGTCTAGCAC<br>AGGGAAGTTTGGCAGGTTCGGAGGAGTCTTCGTGCCGGAGAC<br>CCTCATAACTTGCTTGAACAAGCTCGCGGCTGAATTCAATTTA<br>ATCCTTCATGATCGTGGGTTTCAGGCTGAGCTTAGGACTGCTC<br>TAAGGGATTACGTGGGCCGTGAAACGCCACTGTATTATGCTAA<br>GAGGCTGTCGGATCACTACAGGAACGGCAAGGGAGAGGGCC<br>CCGATATTTACCTAAAGAGGGAAGACCTCAACCACGGCGGCG<br>CACACAAGATCAACAACGCCATTGCTCAGGCCATGATAGCCAA<br>GCGCATGGGCAGAAAGAGAGTAGTGGCCGCCACGGGCGCCG<br>GCCAGCACGGCGTCGCGACGGCCTCGGCCTGCGCTCAGCTC<br>GGATTGGAGTGCACGGTGTTCATGGGCAATGTGGACATGGAG<br>AGACAGCCGTCCAACGTGCTGTTGATGAAGATACTTGGTGCTC<br>AGATAAAGTCAGTTGAAGGATCTTTCAAGGATGCGACATCAGA<br>AGCAATCCGGCACTGGGTCGGAGACCTAGACAACGGCTATTT<br>CTTGACAGGGATGGCGGTGGGGCCGCACCCACTGCCAACCA<br>TGGTTAGAGAGTTCCAAGCTGTGATTGGTAAGGAAACCCGTC<br>GACAAGCGAGGGAGAAGTGGGGAGGCAAGCCGGATGTTGTG<br>GTCGCYTGCGTGGGGAGTGGATCCAATGCTCTCGGCATATTT<br>CATGAGTTTGTCAAGGATGAAGACGTCCGGCTAATCGGCGTA<br>GAGGCCGCCGGGAGCGGAATAGACACCGGTAAACATTCAGCT<br>ACTCTGTCAACAGGAGACGTAGGAGTGTACCATGGATCAATG<br>AGCTATTTGTTGCAAGATGATGAGGGCCAAATCATTGGCCCAC<br>ATTCCATAGGCGTCGGGCTGGAGTATCCCGGCGTTAGTCCAG<br>AGCTGAGCTTTCTTAAAGATATAGGGCGTGCCGAGTTTCACAC<br>GGTCACGGATGAAGAAGCCCTCGACGCATACGCAGTGCTGTG<br>CCGATTAGAAGGGATAATTCCGGCACTCGAAGCAGCCCATGC<br>TTTGGCCTACCTGGGAAAGCTTTGCAAGACGCTGCCCGACGG<br>CGCAAAGGTTGTCGTTAATTGCAGCGGCCGAGGGGATAAGGA<br>TGCCGCCACTGTTTTCAACCACCAACAGCAACAGAAACAATAA
Nicotiana benthamiana TSB-like	PQ246689	ATGGCGTGTAACAAGGATGTTATTTTTGGACAAGCAAAATTTG<br>CTACATCTGGTGAACTAAGATTGTCATATAGCAGAAAATGGAA<br>AGGAAAATTTGCAATATTTTCCTTAGCTACTGGCCCGAGCCGA<br>GTTACAGAGTTACCAAGGAAGCTGGTTTACCATGAAAAAAAGA<br>GAGCAATATTTAGCAATGAAAAGTTTGGGATTTTTGGTGGAAA<br>GTTCGTGCCTGAGACGCTTATATCTTCTTTAACAAAGCTTGAAT<br>ACGAATTCAACTCTGCTTTGCATGACCTTCAGTTTCAGATAGA<br>GCTCGGAGTGGCACTAAGGGACTACGTAGGCCGTGAAACTCC<br>TTTATACTTCGCCGAGCGACTAACAGATTACTACAAGAGCATA<br>AATGGGGGAAAAGGGCCAGACATCTATCTAAAAAGAGAAGAT<br>CTGAACCATGTTGGAGCACACAAGATTAACAACGCTGTTGCAC<br>AAACAATGTTGGCTAAGCGTATGTACTGTAAACATATCATAGC<br>AGCCACCGGTGCGGGCCAGCACGGCATCGCGACGGCGGCTG<br>CCTGTGCAAAACTCTCAATGGAGTGTACTGTATTCATTGGAAG<br>CTTAGATATGGAGAGACTACCTTCTAATGTACTCTTGATGAAG<br>CATCTTGGTGCAAAGGTCAAATATGTTAAAGGAAGTTTCAAGG<br>ATGCAGTATCGGAAGGCATTCGACATTGGGTTAACAACTTGGA<br>GACAAGCTATTTCTTAGCAGGTGCAGCCATAGGACCACACCC<br>ATGTCCAACCATGGTTCGTGAATTCCAATCAGTGATTGGAAAA

		GAAACGAGGAAACAAGCAATGGACAAATGGGGTGGGAAACCA<br>GATGTGTTAGTGGCTTGTGTAGGTAGCGGCTCTAATGCGTTG<br>GGTCTATTTCATGAATTTATTGAAGATGAAGATGTGAGACTAAT<br>AGGAGTTGAAGCTGGTGGGATAGGTCTTGATACAGGTAAACA<br>CTCAGCAACTATGGCTAGAGGTCATGTTGGAGTGTACCATGG<br>AGCCATGTCCTATCTATTACAGGATGAGGAAGGACAAATTATT<br>GAACCACACTCAATAGGCGTGGGATTAGAGTACCCAGGTGTT<br>AGCCCAGAGATTAGCTTTCTGAAAGACATAGGGCGCGCAGAG<br>TTTTCTACAGTCACAAATGAACAAGCCTTAGAAGCATATAAACG<br>GCTGTGCAGACTAGAAGGGATATTCCCAGCCTTAGAAGCTTCT<br>CACGCACTTGCATTTCTTGACAAACTTTGCCCTACTCTAGAGG<br>ATGGTGAGAAGGTGGTTGTTAATTTAAGTGGCCGTGGAGATAA<br>GGATGCTGCCATAGTCTTCAATCATACAACAAAACATGAATAA
Populus trichocarpa TSB-like	PQ246690	ATGGATAAGACTTGCAGTATCCACACAACTTTCTTGAATCGCC<br>AATTACACGGTGGTTTATGTCCTGCCACCAAGCACATAAGCCT<br>AATAACAACTGATAAGAGAAGAACTGGAAGTGGCCAAACAGTA<br>CTCTGTTCACTTGTTACTCCTAAAACTATGAATACGAGGACATT<br>GATTGAAAGACAGGTTCCCCGTGAGGTGTCAATGCCAGCAAA<br>ATTTGGCATGTTTGGAGGGAATTTTGTGCCTGAGACCCTAATC<br>ACTAGTTTGAAGAAGCTGGAAGCTGAATTCATATATGCTTTACA<br>GGATACTGAATTTCAGGAGGAGCTTGCGACGGCCCTGAGAGA<br>CTACGTAGGAAGGGAAACACCTCTGTATTTTGCTCAAGGATTG<br>ACAAATTACTACAAGAATAAAGATGGAGAAGGGGCAGAAATTT<br>ACCTTAAGAGGGAGGATCTCAATCATTGTGGAGCACACAAGAT<br>GAACAACGCTGTTGCGCAGACGATGATTGCCAAACGCATGGG<br>CCTGAAAAGTGTTGTGGCAGCCACTGGTGCTGGACAACATGG<br>TGTTGCAACTGCAGCTGCATGTGCCAAGCTGTCTTTGTCTTGC<br>ACTGTCTTCATGGGCTCTGATGATATGGAAAAACAATCCTCAA<br>ACGTACTGTTAATGAAACTATTTGGTGCTCAGGTTAAACCTGT<br>GGAAAGAAGTTTTAAAGAAGCAAGCTCAGAGGCAATCAGGGA<br>ATGGGCAAGTAACTTAGAGACAAGCTACTACCTGACAGGCAC<br>GGCCGTGGGGCCTCATCCATGTCCAAGCATGGTCTGTGAATT<br>TCAGTCTGTGATTGGAAAGGAGACAAGGAGGCAAGCAATGGA<br>GAAATGGGGTGGCAAGCCAGATGTGTTGATTGCCTGTATCGG<br>GAGTGGGTCTAATGCAATGGGGTTGTTCCATGAATTCATAGAA<br>GATGAAGATGTGAGGTTGATAGGAGTTGAGGCTGCAGGGTTT<br>GGCTTGGATAGTGGGAAACATGCTGCAACTCTGAGTAAAGGA<br>GAAGTTGGGGTCTATCATGGAGCCATGAGCTATTTGTTGGAAG<br>ATGAAGAAGGACAAATAATACGACCACACTCAATCGCTGTGGG<br>GCTGGAATATCCAGGGGTTAGTCCGCAGCTGAGCTTTCTTAAG<br>GACACAGAACGTGCTGAGTTTTATTCTGCCACAGATAAAGAGG<br>CTGTAGATGCATACCTAAGGCTAAGCAGATTAGAAGGCATAAT<br>TCCATCCTTGGAGGCAGCTCATGCACTGGCATACCTCGACAA<br>AGTTTGTCCTACTTTACCAAATGCCACCAAAGTCATAGTAAATT<br>GTAGCGGTCGAGGAGATAAAGATGCAGGAATGGTTCTTGACC<br>ACAGACTTGAGATAAATTAA
Arabidopsis thaliana TSB-like	PQ246691	ATGTCGTCCAGTAAAATCCAGGTACGAGGGCAGCCACTTCTTA<br>GGGTTCCGGCAAGAAACCACCGTATGACACACTTGGTTGTTTG<br>CGGCGTTTCCACAAAACGCCACCACCGTGAAATCAATGCACT<br>CAGCAGTAATAGTGGTCCATCGTTGGATTCGGTCCCAACAAGA<br>ACCGACAAGAGGCAGTTTCTTCGTGGTGACGGTAACGGTAAA<br>TTCGGGAGGTTCGGTGGGAAGTTTGTACCGGAGACATTGATG<br>TCTCGCCTGATAGAACTTGAAGACGAATTTAACTTTGTTAGGT<br>GCGATCACGAATTTCAGGAGGAGCTTACCACAGCGTTAAGAG<br>ACTACGTAGGAAGAGAAACGCCTCTCTACTTCGCCGAACGTCT<br>AACCGAACACTACAAGAACATAGTTCCAACCATCGAAGGTGGA

		CCAGAGATATACTTGAAACGGGAAGATCTTAGCCACTGTGGGT<br>CTCACAAAATCAATAATGCTCTCGCTCAGGCCATGATTTCCCG<br>GCGGCTTGGTTGCAGCCGTGTGGTGGCGGCCACTGGAGCCG<br>GACAACATGGAGTCGCCACAGCGGCTGCATGTGCAAAGCTCT<br>CCTTGGAGTGTACTGTTTTCATGGGAGCTGCTGATATAGAGAA<br>ACAATCCTTTAATGTACTTTCCATGAAACTCCTTGGTGCTCAGG<br>TTATATCAGTGGAAGGAACATTCAAAGATGCAAGTTCAGAAGC<br>TATACGAAACTGGGTCGAAAACCTATATACCACATACTACTTAT<br>CGGGTACGGTCGTAGGACCGCATCCGTGTCCGATAATAGTAC<br>GTGAGTTTCAATCTGTGATCGGGAAAGAGACAAGAAGGCAAG<br>CCAAGCAACTATGGGGTGGTAAGCCTGATGTGTTGGTGGCCT<br>GTGTGGGAAGTGGCTCAAACGCATTGGGTTTGTTCCATGAGTT<br>TGTTGGGGATGAGGATGTGCGGTTAGTCGGTGTCGAGGCTGC<br>GGGGCTTGGTCTGGATTCGGGGAAACATTCAGCTACTTTGGC<br>CTTTGGAGATGTTGGTGTATACCATGGTTCCATGAGCTATTTAT<br>TGCAAGATGATCAAGGACAGATACTTAAACCACACTCCGTAGG<br>TGTTGGGTTAGAGTATCCCGGAGTTGGACCGGAGATTAGTTTC<br>ATGAAAGAAACTGGCAGAGCGGAGTTCTATACAGCAACAGAC<br>GAAGAAGCAATTCAAGCGTGCATGCGATTAAGCAGATTGGAG<br>GGAATAATACCGGCATTAGAAGCGTCTCACGCGCTCGCGTTC<br>CTCGACAAACTCGTGCCTACTCTTCGTGATGGAGCCAAGGTG<br>GTAGTGAACTGCAGTGGCCGTGGTGACAAAGATTTAGATACTC<br>TCATCCAACGAGGCATGCCTTCTTCTTTTTGTTGA
Lamium galeobdolon TSB-like	PQ246692	ATGTCTGGCATCAAAATTATCAACAGTACTAGTGTATCTTCGCA<br>TCTGCAGGGACAAATTTGTCCAAAAATGCCACAAAGCAATTGG<br>CCAACGGTTGGAGATAAACGTTCCAGAATCTCCTGCGTGATGA<br>CTCAACCTAAAAATCGTCATCCCACACCAAAATCAGACCTTCTT<br>TGCCTCATAGATCAAAACCAAGAATCATCGCCAAGTACAGGAA<br>AATTTGGAGGATTTGGAGGAGTCTTCGTGCCCGAAACACTCGT<br>CACTTGCCTCACCAACCTCGCAATTGAGTTCAAACTACTCATG<br>CGTGATCCCCACTTTCAGGAGGAGCTTGCGATTGGTCTAAGA<br>GATTACGTGGGTCGTGAAACGCCTCTTTATTTTGCTCAAAGGC<br>TGACCGACCATTACAAGAATGATAAAGGAGAAGGGCCGGAGA<br>TTTACTTGAAGAGGGAAGATCTCAACCATGGTGGGGCTCACAA<br>GATTAACAACGCCCTTGCTCAAGCCATGCTCGCCAAACGAATG<br>GGTCGGAAAAGCGTCGTGGCGGCCACAGGCGCCGGCCAGCA<br>CGGCGTCGCAACAGCCGCCGTTTGTGCTAAGCTTGACTTGGA<br>ATGTACAATAATCATGGGCAAGCTGGATATGGAGAGACAGCC<br>CTCAAACGTGCTCCTAATGAAGCACCTTGGCGCTCAGGTGAA<br>AAGTGTTGAAGGGACGTTCAAGGATGCGACGTCGGAAGCAAT<br>CCGGGTGTGGGTGGGAGACTTAGAGGAGAGCTACTACTTGGC<br>CGGGACGGCGGTGGGGCCGCATCCGTGCCCGAGCATGGTCC<br>GGGAGTTCCAATCGGTGATCGGAAAAGAAACGAGGAAACAGG<br>CCATGGAGAAGTGGGGCGGGAAGCCGGACGTGGTGGTCGCC<br>TGCGTGGGAAGCGGGTCGAATGCTCTGGGAATATTTCATGAA<br>TTCGTGAGGGATGAACAAGTGAGGTTGATTGGAGTTGAAGCT<br>GGTGGGACCGGAATAAATGGCGATAAACATTCAGCAACTCTG<br>GTTAAAGGAGAGGTGGGTGTGTACCATGGGGCCATGACCTAT<br>TTGTTGCAAGATGATGAGGGCCAAATCATTTGCCCACACTCCA<br>TAGGTGTTGGGCTAGAATACCCGGGGGTTAGTCCAGAGCTGA<br>GCTTTCTCAGAGACATTGGGCGTGCAGAGTTCTACGCCGTCA<br>CAGATGAAGAAGCTCTTGATGCATACTCGTTGTTGTGCCGATT<br>AGAAGGTATATTTCCAGCTCTAGAATCTGCTCATGCATTGGCG<br>TATCTTGGAAGACTTTGCCGGACATTGCCTAATGGCGCCAAGG<br>TGGTGGTTAATCTCAGTGGCCGGGGCGATAAGGATGCACACA<br>CAGTGTTTAAGTATCAACAACACAAGAATACCTAA

Aphelandra squarrosa TSB	PQ246693	ATGCCCGCGTCTGCAGCGGCTTCGCGATTCATCTCCTGCGGC<br>GCCGCCCAGGCCGACCTCCCGTCGCAGCTCAATCTCCAATCC<br>CTTTTGCCCCTCAAATCCAGTCGGATTGCGCCCACCGGCCCA<br>TTCGATTCTCGTCGCCCTCTCGTTGTTTGTGCGGTCAACATGG<br>CGGCGTCGGCGGTGACGGCGGTTGAGAAGGAAACCGCGGCG<br>GCGGGGGAGGCGCTGCAGCGGCCCGATTCGTCCGGAAGATA<br>TGGAAAATTTGGGGGCAAGTACGTTCCCGAGACCCTGATGTA<br>TGCGCTGTCGGAACTCGAGGCGGCGTTCAAAGCCCTCTCCAA<br>TGACGATGAATTTCAGAAAGAGCTATCAGGAATATTGAAAGAC<br>TATGTTGGACGAGAAAGCCCGCTTTACTTTGCTGAACGTCTTA<br>CTGAGCACCATAAGCGTGCGGATGGGACAGGGCCTCACATAT<br>ATCTCAAAAGGGAAGACCTTAACCACACTGGTGCACACAAAAT<br>CAACAATGCTGTTGCACAAGCTTTGCTTGCAAAGCGTCTCGGT<br>AAGAAGCGTATCATTGCAGAGACCGGAGCTGGTCAGCATGGT<br>GTAGCAACAGCTACGGTTTGTGCGCGGTTTGGGTTGCAATGT<br>ATCATATATATGGGTGCTCAAGATATGGAGAGGCAAGCACTTA<br>ATGTCTTCCGAATGCGGTTGCTTGGCGCTGAGGTTAGAGGAG<br>TTCATTCTGGGACGGCCACACTGAAGGATGCTACATCTGAAG<br>CTATCCGAGATTGGGTGTCTAATGTTGAAACTACTCATTACATA<br>TTGGGGTCTGTTGCTGGACCACATCCGTATCCTATGCTGGTAA<br>GGGATTTTCATGCAGTGATTGGTAAGGAAACCAGAAGACAGG<br>CTTTGGAAAAATGGGGTGGTAAACCTGATGTGCTCGTTGCTTG<br>CATTGGTGGAGGTTCAAATGCGATGGGGCTCTTTCACGAGTTT<br>ATTGATGACAAGGATGTTAGGTTAATTGGCGTTGAGGCTGCTG<br>GGTTTGGTTTAGATAGTGGTAAGCATGCTGCCACTCTGAGTAA<br>AGGAGAAGTTGGAGTTCTTCATGGAGCCATGAGTTACTTGTTG<br>CAAGATGAAGATGGGCAAGTAATTGAGCCTCATTCTATAAGTG<br>CTGGCCTGGACTACCCTGGAGTTGGACCTGAGCACAGCTTCC<br>TGAAAGACATTGGACGGGCTGAGTATCATAGCCTCACTGATGA<br>GGAGGCCTTACAAGCTTTTAAGAGGTTATCTCGGCTAGAGGG<br>AATTATCCCTGCACTGGAGACATCCCATGCTCTTGGTTATCTA<br>GAGTACCTATGCCCGACTCTTCCCGATGGGACTAAGGTTGTG<br>GTGAACTGCAGCGGGAGAGGTGATAAAGATGTCCACACAGCC<br>CTCAAGTATTTGAATATGTAA
Aphelandra squarrosa TSB type II	PQ246694	ATGGCGCAATCTGTCTTCCTCACCCCTTCTGCCAACGCACGGT<br>GTTCCATTCAAGGATACAAACACCAGTTGGGTTATTTTGCTTC<br>GAAGGAAAAGCCATGCCATCTGAAACTCGTGCCCAAATCAAGT<br>TTATCTCCCACAGCAGCCCAAATTTTCAATTCCCGTGCAATCG<br>AAATCCCCCGCCAGTGGTACAACCTAGTTGCAGATCTTCCCGT<br>AAAGCCCCCACCTCCCTTGCATCCCAAGACTTTCGAACCCGTA<br>AAACCAGACGACCTGACGCCTTTATTCCCCGATGAATTAATCA<br>AGCAGGAGGCCACCCTCGAAAGGTTCATCGACATCCCTGATG<br>AGGTCGTTGACGTTTATCGACTTTGGCGCCCGACGCCTCTCAT<br>CCGGGCTAAGCGGTTGGAAAAGCTGCTCGACACGCCGGCCA<br>GAATCTATTACAAGTACGAAGGCGGCAGCCCGGCCGGTTCGC<br>ATAAGCCGAACTCGGCTGTGCCGCAGGCATGGTACAATGCGC<br>AGCAGGGCGTCAAGAATGTCGTCACAGAGACCGGCGCGGGG<br>CAGTGGGGGAGCTCCCTGGCGTTTGCGTGTAGCTTATTCGGG<br>CTCAACTGTGAGGTGTGGCAAGTTCGTGCTTCGTATGATCAGA<br>AGCCGTATCGGAAACTGATGATGCAAACTTGGGGTGCGAAGG<br>TGCACCCTTCGCCTTCGGACATAACGGAAGCCGGTAGACGAA<br>TACTCGAGCATGATGCGTCAAGCCCGGGGAGTTTAGGAATTG<br>CCATATCCGAGGCTGTGGAGGTTGCAGCTGCGAATCCGGACA<br>CCAAGTATTGTTTGGGCAGTGTTCTTAACCATGTACTGTTGCAT<br>CAAACTGTCATTGGCGAGGAGTGCATAAAGCAGATGGAGGCG<br>ATAGGAGAAACTCCTGATGTGATCATTGGATGCACTGGGGGT

		GGATCCAATTTTGGAGGGCTTGCTTTCCCGTTTATTCGGGAGAAGCTCAATGGGAAGATTAACCCCGTGATTAGAGCAGTTGAACCAGAAGCATGCCCCTCGCTGACCAAGGGGGTGTATGCGTACGATTATGGGGATACTGCAGGGATGACTCCCTTGCTGAAGATGCATACTCTTGGCCATGATTTCATTCCTGATCCAATTCATTCTGGGGGCTTGAGGTACCATGGCATGGCTCCACTGATTTCACATGTCTATGAACTGGGATTCATGGAAACCATGGCAATTGCGCAGACTGAATGCTTTGAGGGGGCGATAAAATTTGCTCGGACTGAGGGGATAATTCCAGCACCAGAACCGACTCATGCTATAGCTGCCACTATAAGGGAAGCGTGTCGTTGCAGAGAGACCGGCGAGTCGAAGGTTATTCTCACAGCAATGTGTGGACATGGCCATTTCGATTTGCCTGCTTACGAGAAGTACCTTCAGGGAGATCTCGTCGACCTCTCCTTCTCGGAGGAGAGCATAAAAGCATCTCTGGCCAAAATTCCTCGGACTTGA
Solanum licopersicum TSB-like	PQ246695	ATGGCATGTAATATTAATGTTGAATCTATTCTTGGACAAGGAATTTTTGCTACTACATCAAGCAAAAAATTACAAGCCTTCCCATCTCATCATACTTATAAAGCTAATGTTATTTCTTGTGTAGCTATTGGCCCAACTCCAATTCCACAATTACCATGGAAGTTAGTTTTCCATGAAAAAGAGAGACAATCATTGTTAAGCAATGAAAAATTTGGGATTTATGGTGGAAAGTTTGTCCCTGAGACACTAATATCTCCTTTAACAAAGCTTGATTATGAATTCAACTCTGCTTTGCGCGATCCTCAATTTCAGATGAATCTTCAAGTAGCATTAAAGGACTATGTAGGACGTGAAACTCCTTTATACTTTGCCCAAAGACTCACAGATTATTACAAGAGTCTTAATAAAGGGATAGGACCAGATATATATCTAAAAAGAGAAGATTTAAACCATGGTGGTGCACACAAAATCAACAACGCTATTGCACAAGCTATGTTGGCTAAGCGTATGGGTTGCAAAAATGTCGTGGCGTCCACCGGTGCTGGACAGCACGGTGTCGCCACGGCGGCTGCTTGTGCCAAACTCTCATTGGAGTGCACAATATTCATGGGAAGTTTAGATATGGAGAGACAACCTTCTAATGTACTCTTGATGAACCATCTTGGTGCAAAGGTGAAATGTGTTGAAGGAAGTTTCAAAGATGCAATGTCAGAAGGTATTAGAAATTGGGTTAACAATTTGGAGACAAGTTATTTCTTAGCGGGTGCAGCCATAGGACCTCACCCATGTCCAACCATGGTTCGTGAATTCCAATCAATTATTGGAAAAGAGACTAGGAAACAAGCCATGGATAAATGGGGTGGCAAACCACATGTGTTGGTGGCTTGTGTTGGGAGTGGCTCCAATGCGTTGGGTTTGTTTCATGAATTTATACAAGATGTAGATGTGAGACTAATCGGAGTCGAGGCCGGCGGGATCGGTCTTGATTCAGGTAAACACTCAGCAACTATGGCTAGGGGTGAAGTTGGAGTGTACCATGGTGCCATGAGCTATTTATTACAAGACGAGGAAGGCCAAATAATTGGACCACATTCCATAGGTGTGGGATTAGAATATCCAGGTGTTAGTCCAGAGCTTAGCTATTTGAAAGATATCGGACGTGCTGAGTTTTCCACCGTAACAGATGAAGAAGCTATAAAAGCATATAAACGATTGTGCATACTAGAAGGGATATTTCCAGCCTTAGAATCTTGTCATGCTCTTGCATTTCTAGACAAACTTTGTTCAACTTTAAAGGATGGTGAAAAAGTGATTGTTAATTTAAGTGGTCGTGGAGATAAGGATGCTGAGGCGGTATTTAATCATACACCAAAACATAAATGA

Supplementary Table 2: List of primers used in this study.

Gene	Vector	Primer Fw	Primer Rv
Zea mays Bx1	3Ω1	TGTTGTTTTTATGAATTTTGCAGATGGCTTTCGCGCCCAAAA	CAGACAACCACAACAAGCTCATGGCAGCGCGTTCTT
Zea mays Bx2	3Ω1	TTATGAATTTTGCAGATGGCTGCTCAACTGCATCACG	GACAACCACAACAAGCACCGTCACGCAGCCTGTGGGAC
Zea mays Bx3	3Ω1	TTATGAATTTTGCAGATGGCCCTTGGAGCTGCG	GACAACCACAACAAGCACCGTCAGGAAGCAATCCTTGGAACAAG
Zea mays Bx4	3Ω1	TTATGAATTTTGCAGATGGCTCTCGAAGCAGCGTACG	GACAACCACAACAAGCACCGTCATTTGGGAATTCTAGGAACAAGGT
Zea mays Bx5	3Ω1	TTATGAATTTTGCAGATGGCACTCCAGGCAGCCT	GACAACCACAACAAGCACCGCTAGACGGCCCTAGGAACAAG
Zea mays Bx8	3Ω1	TTTATGAATTTTGCAGCTCGATGGCAGCATCGTGCGGC	GACAACCACAACAAGCACCGTCAGTAGGAGTTTATGAGATGAACC
Consolida orientalis Bx1	3Ω1	TTTATGAATTTTGCAGCTCGATGGCACTTGCAATTACAAGCTCAG	GACAACCACAACAAGCACCGTTAAATCATGAGTAGACTGTTGTTTTC
Lamium galeobdolon TSA-1	3Ω1	TTTATGAATTTTGCAGCTCGATGGCTTCTTCTCTCAAGGCAAC	GACAACCACAACAAGCACCGTCAAGAAAGTGCAGATTTCAAACTTTTG
Lamium galeobdolon TSA-2	3Ω1	TTTATGAATTTTGCAGCTCGATGGCCGCTAATTCTCTCAAGTC	GACAACCACAACAAGCACCGTCAAACAAGTGCAGATTTTAAGGTTTTG
Aphelandra squarrosa TSA	3Ω1	TTTATGAATTTTGCAGCTCGATGGCTGCTGCTGCTCTCAAAG	GACAACCACAACAAGCACCGCTATAACAGAACTGAATCACCCCTC
Aphelandra squarrosa TSB-like	3Ω1	TTTATGAATTTTGCAGCTCGATGTCCTATAGTAAATGTCTTCC	GACAACCACAACAAGCACCGTTATTGTTTCTGTTGCTGTTGG
Nicotiana benthamiana TSB-like	3Ω1	TTTATGAATTTTGCAGCTCGATGGCGTGTAACAAGGATGTTATTTTTGG	GACAACCACAACAAGCACCGTTATTCATGTTTTGTTGTATGATTGAAGAC
Populus trichocarpa TSB-like	3Ω1	TTTATGAATTTTGCAGCTCGATGGATAAGACTTGCAGTATCCACACAAC	GACAACCACAACAAGCACCGTTAATTTATCTCAAGTCTGTGGTCAAG
Arabidopsis thaliana TSB-like	3Ω1	TTTATGAATTTTGCAGCTCGATGTCGTCCAGTAAAATCCAGG	GACAACCACAACAAGCACCGTCAACAAAAAGAAGAAGGCATGC
Arabidopsis thaliana INS	3Ω1	TTTATGAATTTTGCAGCTCGATGGATCTTCTCAAGACTCC	GACAACCACAACAAGCACCGTCAAGAGACAAGAGCAGACTTC
Lamium galeobdolon TSB-like	3Ω1	TTTATGAATTTTGCAGCTCGATGTCTGGCATCAAAATTATCAAC	GACAACCACAACAAGCACCGTTAGGTATTCTTGTGTTGTTGATAC
Aphelandra squarrosa TSB-like	BsaI domestication	GAAACCCTCATAACTTGCTTGAAC	GCAAGTTATGAGGGTTTCCGGCACGAAGACTCC
Aphelandra squarrosa TSB-like	BsaI domestication	GATCTAGACAACGGCTATTTC	ATAGCCGTTGTCTAGATCTCCGACCCAGTGC
Aphelandra squarrosa TSB-like	3α1	ATGTCCTATAGTAAATGTCTTCCCTCC	GCCGCTTTGTTTCTGTTGCT

eYFP	3α1	GTGAGCAAGGGCGAGGAG	TTACTTGTACAGCTCGTCCA
Aphelandra squarrosa TSA	3α1	ATGGCTGCTGCTGCTCTC	GCCGCTTAACAGAACTGAATCA
mCeruleans	3α1	ATGGTGAGCAAGGGCGAG	TTACTTGTACAGCTCGTCCA
Aphelandra squarrosa TSB-like	pOPINF	AAGTTCTGTTTCAGGGCCCGATGACGACTCAGGACGTGC	ATGGTCTAGAAAGCTTTATTATTGTTTCTGTTGCTGTTGG
Aphelandra squarrosa TSA	pOPINF	AAGTTCTGTTTCAGGGCCCGATGGCTGCTCTCACGACTGC	ATGGTCTAGAAAGCTTTACTATAACAGAACTGAATCACCCCTC
Aphelandra squarrosa TSB	pOPINF	AAGTTCTGTTTCAGGGCCCGATGGCGGCGTCGGCG	ATGGTCTAGAAAGCTTTATTACATATTCAAATACTTGAGGGC
Aphelandra squarrosa TSA C-His	pET28	TGTTTAACTTTAAGAAGGAGATATACATGGCTGCTCTCACGACTG	GTGGTGGTGGTGGTGTTCTAACAGAACTGAATCACCCCTCG
Aphelandra squarrosa TSB-like C-His	pOPINE	AGGAGATATACCATGACGACTCAGGACGTG	GTGATGGTGATGTTTTTGTTTCTGTTGCTGTTGGTGG
Aphelandra squarrosa TSA N-His	pET28	AATGGGTCGCGGATCATGGCTGCTCTCACGACTG	TGCTCGAGTGCGGCCTCATAACAGAACTGAATCACCCCT
Aphelandra squarrosa TSB	3Ω1	TTTATGAATTTTGCAGCTCGATGCCCGCGTCTGCAGCG	GACAACCACAACAAGCACCGTTACATATTCAAATACTTGAGGGC
Aphelandra squarrosa TSB type II	3Ω1	TTTATGAATTTTGCAGCTCGATGGCGCAATCTGTCTTCC	GACAACCACAACAAGCACCGTCAAGTCCGAGGAATTTTGGC
Aphelandra squarrosa TSB-like A195E	mutagenesis primers	GAGACGGGCGCCGGCCA	TGGCCGGCGCCCGTCTCGGCCACTACTCTCTTTCTGC
Aphelandra squarrosa TSB-like E388D	mutagenesis primers	GATTATCCCGGCGTTAGTCCAGAG	ACTAACGCCGGGATAATCCAGCCCGACGCCTATGG
Lamium galeobdolon TSA-1	pOPINF	AAGTTCTGTTTCAGGGCCCGATGGCCACTCTCACCGCC	ATGGTCTAGAAAGCTTTATCAAGAAAGTGCAGATTTCAAACTTTTG
Lamium galeobdolon TSA-2	pOPINF	AAGTTCTGTTTCAGGGCCCGATGGCTACTCTCCAAACTGC	ATGGTCTAGAAAGCTTTATCAAACAAGTGCAGATTTTAAGGTTTTG
Lamium galeobdolon TSB-like	pOPINF	AAGTTCTGTTTCAGGGCCCGATGACTCAACCTAATATTCGTCATCC	ATGGTCTAGAAAGCTTTATTAGGTATTCTTGTGTTGTTGATAC
Nicotiana benthamiana TSB-like	pOPINF	AAGTTCTGTTTCAGGGCCCGTCCTTAGCTACTGGCCCGAGC	ATGGTCTAGAAAGCTTTATTATTCATGTTTTGTTGTATGATTGAAGAC
Nicotiana benthamiana TSA	pOPINF	AAGTTCTGTTTCAGGGCCCGATGGCTGCCCTCAGCACC	ATGGTCTAGAAAGCTTTATCAAGAGAGTGCAGATTTTAAAGATGTC
Populus trichocarpa TSB-like	pOPINF	AAGTTCTGTTTCAGGGCCCGATGAATACGAGGACATTGATTGAAAG	ATGGTCTAGAAAGCTTTATTAATTTATCTCAAGTCTGTGGTCAAG
Populus trichocarpa TSA	pOPINF	AAGTTCTGTTTCAGGGCCCGATGGCGTCTCTCACTGCAACC	ATGGTCTAGAAAGCTTTATCAAGGAAGCGCAGCTTTCAAAG
Arabidopsis thaliana INS	pOPINF	AAGTTCTGTTTCAGGGCCCGATGGATCTTCTCAAGACTCC	ATGGTCTAGAAAGCTTTATCAAGAGACAAGAGCAGACTTC

Arabidopsis thaliana TSA	pOPINF	AAGTTCTGTTTCAGGGCCCGATGGCTTCTCTCTCCACCTCTTC	ATGGTCTAGAAAGCTTTATCAAAGAAGAGCAGATTTAAGAGAC
Arabidopsis thaliana TSB-like	pOPINF	AAGTTCTGTTTCAGGGCCCGGTCCCAACAAGAACCGACAAG	ATGGTCTAGAAAGCTTTATCAACAAAAAGAAGAAGGCATGC
Solanum licopersicum TSB-like	3Ω1	TTTATGAATTTTGCAGCTCGATGGCATGTAATATTAATGTTGAATCTATTCTTG	GACAACCACAACAAGCACCGTCATTTATGTTTTGGTGTATGATTAAATACCG
Aphelandra squarrosa TSB E190A	mutagenesis primers	GCCACCGGAGCTGGTC	TGCTACACCATGCTGACCAGCTCCGGTGGCTGCAATGATACGCTTC
Aphelandra squarrosa TSB D386E	mutagenesis primers	GAGTACCCTGGAGTTGGACCTG	TCCAACTCCAGGGTACTCCAGGCCAGCACTTATAG
Nicotiana benthamiana EF1a_qP	qPCR	ATTGGTACTGTCCCTGTCGG	CGTGGTGCATCTCAACAGAC
Nicotiana benthamiana PP2A_qP	qPCR	GAATTTGGTCCAGAGTGGGC	GGTGCAAGCAATGAAATCGC
Nicotiana benthamiana TSA_qP	qPCR	GTTGTGCCTCAGTTGTCCTG	CAAGTCCATGTACACCAGCAT
Nicotiana benthamiana TSBlike_qP	qPCR	GCCTTAGAAGCTTCTCACGC	ATCCTTATCTCCACGGCCAC

Supplementary References

1 Rowlett, R. *et al.* Mutations in the Contact Region between the α and β Subunits of Tryptophan Synthase Alter Subunit Interaction and Intersubunit Communication. *Biochemistry* 37, 2961–2968 (1998).

2. Almhjell, P. J. *et al.* The β-subunit of tryptophan synthase is a latent tyrosine synthase. *Nat Chem Biol* 1–8 (2024), doi:10.1038/s41589-024-01619-z.

3. Coolen, S. et al. Transcriptome dynamics of Arabidopsis during sequential biotic and abiotic stresses. Plant J 86, 249–267 (2016).

4. Irmisch, S., Jiang, Y., Chen, F., Gershenzon, J. & Köllner, T. G. Terpene synthases and their contribution to herbivore-induced volatile emission in western balsam poplar (*Populus trichocarpa*). BMC Plant Biol 14, 270 (2014).

5. D'Esposito, D. et al. Tomato transcriptomic response to *Tuta absoluta* infestation. BMC Plant Biology 21, 358 (2021).

6. Heil, M. et al. How Plants Sense Wounds: Damaged-Self Recognition Is Based on Plant-Derived Elicitors and Induces Octadecanoid Signaling. PLOS ONE 7, e30537 (2012).
