# Supplementary material for: A pseudoenzyme enables indole biosynthesis in eudicot plants
Source: Nat Chem Biol. 2025 Jun 25;22(1):120–7. doi: 10.1038/s41589-025-01943-y (PMC12727527; doi:10.1038/s41589-025-01943-y)
Supplement: Supplementary file 7 — Unprocessed SDS–PAGE and western blot. [file 41589_2025_1943_MOESM7_ESM.pdf]

A

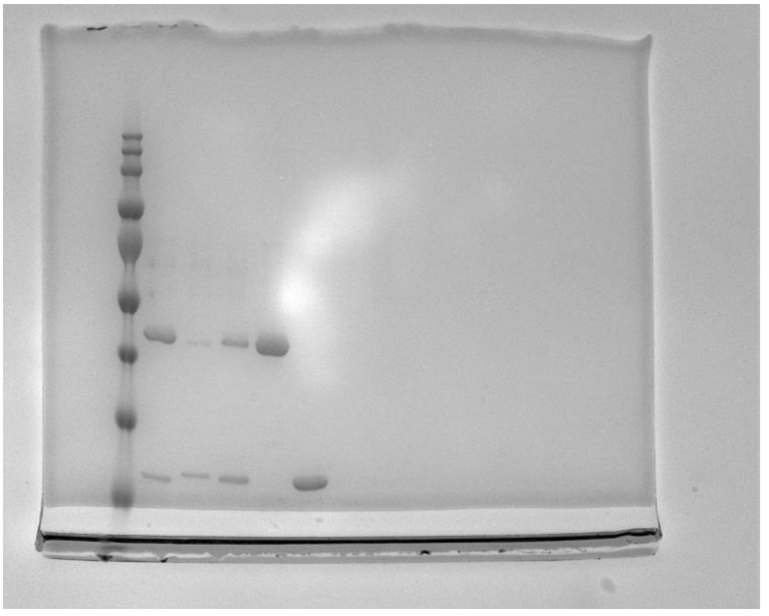

B

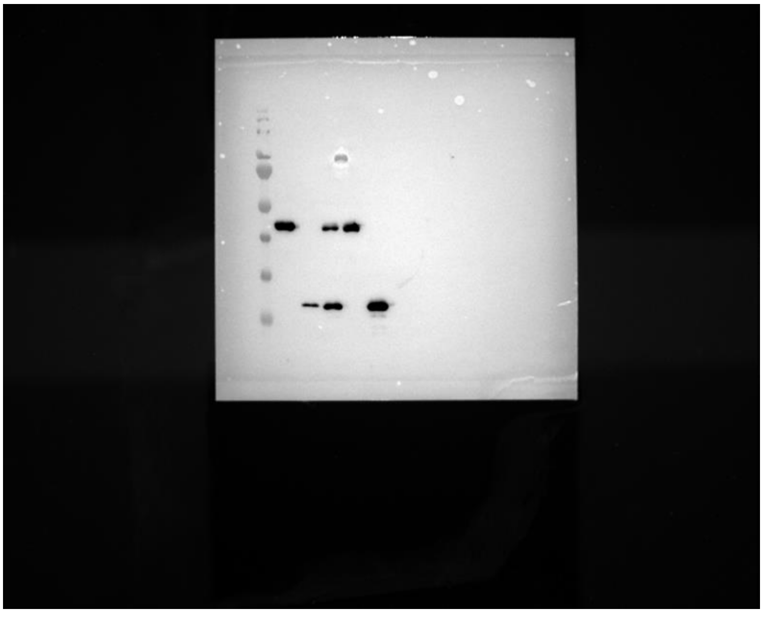

Source Data File for Figure 3C: A) Uncropped image of SDS-page. **B**) Uncropped image of Western blot. Illuminated image of the membrane was superimposed on luminescence image for protein marker alignment.
